# Spontaneous gain of susceptibility suggests a novel mechanism of resistance to hybrid dysgenesis in *Drosophila virilis*

**DOI:** 10.1371/journal.pgen.1007400

**Published:** 2018-05-29

**Authors:** Sergei Y. Funikov, Dina A. Kulikova, George S. Krasnov, Alexander P. Rezvykh, Lubov N. Chuvakova, Natalia G. Shostak, Elena S. Zelentsova, Justin P. Blumenstiel, Michael B. Evgen’ev

**Affiliations:** 1 Engelhardt Institute of Molecular Biology, Russian Academy of Sciences, Moscow, Russia; 2 Koltzov Institute of Developmental Biology, Russian Academy of Sciences, Moscow, Russia; 3 Department of Ecology and Evolutionary Biology, University of Kansas, Lawrence, KS, United States of America; Cornell University, UNITED STATES

## Abstract

Syndromes of hybrid dysgenesis (HD) have been critical for our understanding of the transgenerational maintenance of genome stability by piRNA. HD in *D*. *virilis* represents a special case of HD since it includes simultaneous mobilization of a set of TEs that belong to different classes. The standard explanation for HD is that eggs of the responder strains lack an abundant pool of piRNAs corresponding to the asymmetric TE families transmitted solely by sperm. However, there are several strains of *D*. *virilis* that lack asymmetric TEs, but exhibit a “neutral” cytotype that confers resistance to HD. To characterize the mechanism of resistance to HD, we performed a comparative analysis of the landscape of ovarian small RNAs in strains that vary in their resistance to HD mediated sterility. We demonstrate that resistance to HD cannot be solely explained by a maternal piRNA pool that matches the assemblage of TEs that likely cause HD. In support of this, we have witnessed a cytotype shift from neutral (*N)* to susceptible (*M*) in a strain devoid of all major TEs implicated in HD. This shift occurred in the absence of significant change in TE copy number and expression of piRNAs homologous to asymmetric TEs. Instead, this shift is associated with a change in the chromatin profile of repeat sequences unlikely to be causative of paternal induction. Overall, our data suggest that resistance to TE-mediated sterility during HD may be achieved by mechanisms that are distinct from the canonical syndromes of HD.

## Author summary

Transposable elements (TE) can proliferate in genomes even if harmful. In response, mechanisms of small-RNA silencing have evolved to repress germline TE activity. Syndromes of hybrid dysgenesis in *Drosophila—*where unregulated TE activity in the germline causes sterility—have also revealed that maternal piRNAs play a critical role in maintaining TE control across generations. However, a syndrome of hybrid dysgenesis in *D*. *virilis* has identified additional complexity in the causes of hybrid dysgenesis. By surveying factors that modulate hybrid dysgenesis in *D*. *virilis*, we show that protection against sterility cannot be entirely explained by piRNAs that control known inducer TEs. Instead, spontaneous changes in the chromatin state of repeat sequences of the mother may also contribute to protection against sterility.

## Introduction

Transposable elements are selfish elements that have the capacity to proliferate in genomes even if they are harmful [1]. In response to this threat, mechanisms of small-RNA based silencing have evolved to limit TE proliferation. In the germline of animals, Piwi-interacting RNAs (piRNAs) function to maintain TE repression through both transcriptional and post-transcriptional silencing [2]. Critically, the epigenetic and transgenerational nature of piRNA-mediated TE control has been revealed by syndromes of hybrid dysgenesis (HD) [3,4]. HD is a syndrome of TE-mediated sterility that occurs when males carrying active copies of TEs are crossed with females where such copies are rare or absent [5–7].

The hybrid dysgenesis syndrome (HD) is defined as a combination of various genetic disorders such as genic mutations and chromosomal aberrations that lead to sterility in the progeny of intraspecific crosses [5–7]. Sterility during HD is mediated by mobilization of certain TE families carried by the paternal genome and absent in the maternal genome [6,7]. To date, there are several independent HD systems in *Drosophila melanogaster*. The most well described are the *I-R* and *P-M* systems, controlled by the *I-element* (a non-LTR (long terminal repeat) retrotransposon) and the *P-element* (a DNA transposon), respectively [6–8]. Activation of paternally inherited TEs is explained by the fact that only the female maintains transgenerational TE repression via piRNAs transmitted through maternal deposition. When the female genome lacks certain TE families, female gametes also lack piRNAs that target these families. Thus, TE families solely transmitted through the male germline become de-repressed in the absence of repressive piRNAs inherited from the mother [2–4,9].

HD in *D*. *virilis* was initially observed when males of laboratory strain *160* and females of wild-type strain *9* were crossed. The F1 progeny exhibited up to 60% sterility, while sterility in the progeny of reciprocal crosses did not exceed 5–7% [10]. Similar to the *D*. *melanogaster P-M* system, the sterility of hybrids from dysgenic crosses is apparently the result of abnormal development (atrophy) of male and female gonads [10–12]. By analogy with the *P-M* system, strain *160* and strain *9* were called “*P*-like” (*P*) and “*M*-like” (*M*), respectively.

In contrast to *I-R* and *P-M* systems, the study of HD in *D*. *virilis* has demonstrated that multiple unrelated TEs belonging to different families are mobilized in dysgenic progeny [13–16]. The TEs presumably causal of dysgenesis and absent in *M*-like strain *9* include *Penelope* (a representative of the *Penelope*-like element (PLE) superfamily), *Paris* and *Polyphemus* (DNA transposons), as well as a non-LTR retrotransposon *Helena* [13–16]. A typical *M*-like strain *9* contains only diverged inactive remnants of these TEs. Additionally, piRNAs targeting *Penelope*, *Paris*, *Polyphemus* and *Helena* are highly abundant in the germline of strain *160* and are practically absent in strain *9* [17,18]. Thus, it has been suggested that the combined activity of these four asymmetric TEs, present only in strain *160*, underlies gonadal atrophy and other manifestations of HD in *D*. *virilis*. This large asymmetry in TE abundance between strains suggests that HD in *D*. *virilis* may be considered a model for understanding the consequences of intermediate divergence in TE profiles within a species.

Nonetheless, recent studies have called into question whether the standard model of HD–described in *D*. *melanogaster* where sterility is caused by the absence of maternal piRNAs that target specific inducing TE families—applies in *D*. *virilis* [3,4,18,19]. This is because several “neutral” (*N*) strains exhibit “immunity” to HD in dysgenic crosses but lack maternal piRNA corresponding to *Penelope* elements, the presumptive primary driver of dysgenesis [19]. If *Penelope* is a key driver of dysgenesis, how do neutral strains exhibit "immunity" in the absence of maternally transmitted *Penelope* piRNA? Two fundamental issues arise. First, as observed in *D*. *melanogaster*, is there a single major element that serves as a key driver of HD in *D*. *virilis*? Second, do *N-*strains confer their resistance to HD solely through maternally provisioned piRNA or through alternate mechanisms? Despite significant progress in understanding the morphogenetic events occurring during gametogenesis and embryogenesis in the progeny of *D*. *virilis* dysgenic crosses, these questions still need to be answered [11,18].

To answer these questions, by using small RNA deep-sequencing and qPCR, we decided to perform a comparative survey of maternal piRNA profiles across several “neutral” strains of different origin that did not quite fit the HD paradigm developed in the previous studies of this phenomenon [3,4,9,19]. Additionally, we developed transgenic strains containing a presumptive causative TE and did not detect a cytotype change after its propagation in the genome. The accumulated data failed to pinpoint a single TE or specific set of TEs responsible for their “immunity” and support a model in which resistance to TE-mediated sterility during dysgenesis may be achieved by a mechanism that varies across strains. We thus propose an alternate model to explain resistance to TE mediated sterility in *D*. *virilis*. Instead of solely being explained by maternal piRNAs that target inducing TE families, the chromatin profile of repeats in the maternal genome may confer general immunity to the harmful effects of TE mobilization.

## Results and discussion

### A survey of neutral strain piRNA profiles reveals that there is no common pattern of maternal piRNAs derived from asymmetric TEs

**Fig 1:**
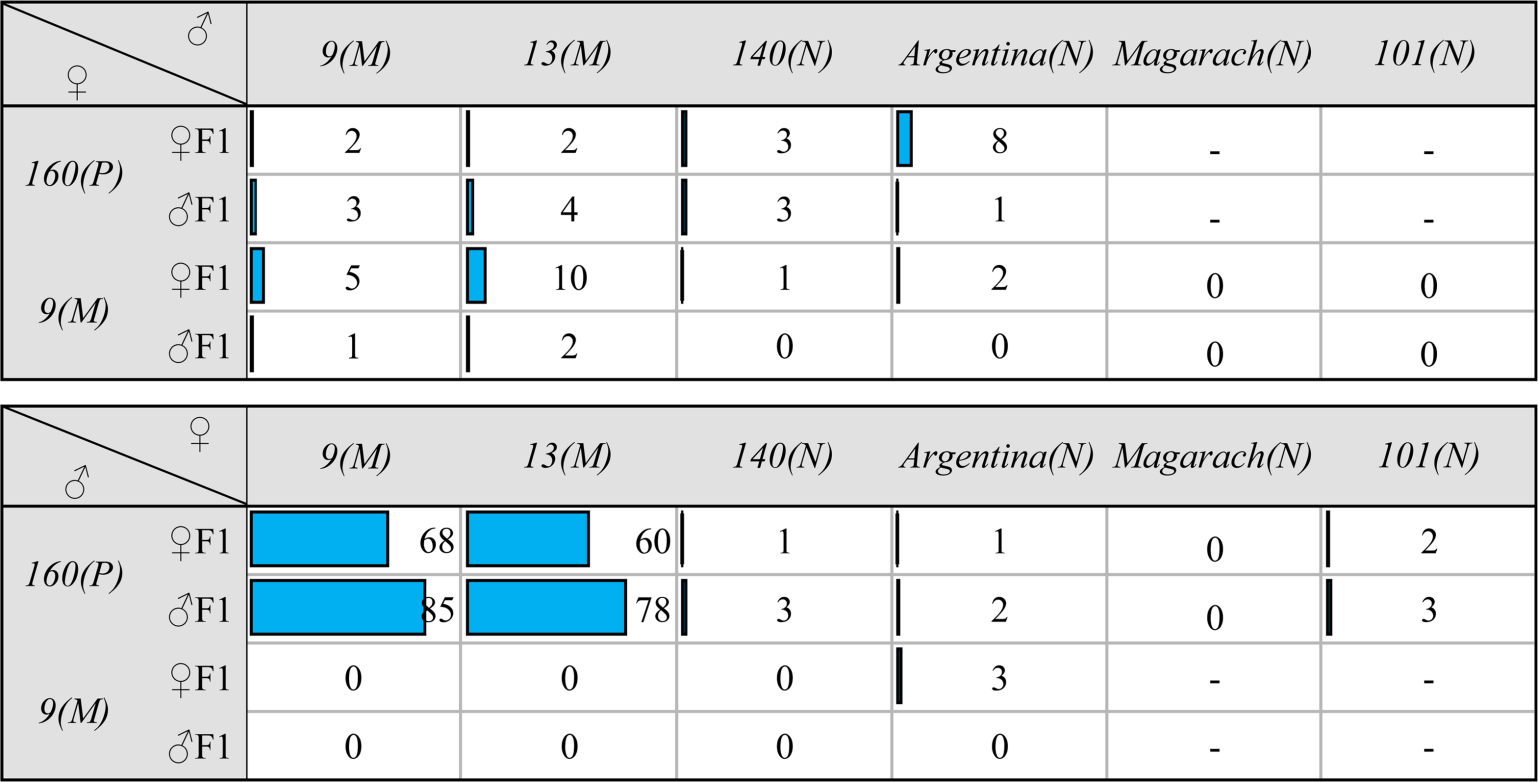
The frequency (in %) of gonadal atrophy in the progeny of dysgenic and reciprocal crosses among the studied *M*-like and neutral strains. The number of examined individuals was ~100–130 separately for females and males.

To characterize the piRNA profiles across diverse strains that vary in resistance to HD, we performed small RNA sequencing on six *D*. *virilis* strains obtained from various sources (see Materials and Methods) and maintained in our laboratory for more than 20 years. These strains exhibit different levels of gonadal atrophy when crossed with males of *P*-like strain *160*. Two of them (*9* and *13*) represent strong *M*-strains (they exhibit up to 65% of gonadal atrophy in the F1 progeny of the dysgenic cross) and four (*140*, *Argentina*, *Magarach* and *101*) behave as “neutral” or *N*-strains when crossed with strain 160 males and, hence, did not exhibit gonadal atrophy (less than 10% atrophied gonads) in such crosses (Fig 1).

Previous studies suggest *Penelope* element as a key driver of HD in *D*. *virilis* [15,20,21]. However, while *N-*strains *140* and *Argentina* both carry *Penelope* elements, two other *N-*strains–*Magarach* and *101* contain neither functional *Penelope* copies nor *Penelope*-derived small RNAs [19]. This observation questions the key role of *Penelope* as a factor determining HD in *D*. *virilis* and suggests that piRNAs targeting other asymmetric TEs, e.g. *Polyphemus*, *Helena* and possibly *Paris*, may provide immunity to HD [14,15,17,21,22]. To explore this possibility we performed a comparative analysis of both classes of small RNAs (piRNAs and siRNAs) in the ovaries of all selected *M*- and *N*-strains using the extended list of TEs and other repeats recently defined in *D*. *virilis* genome [18].

**Fig 2:**
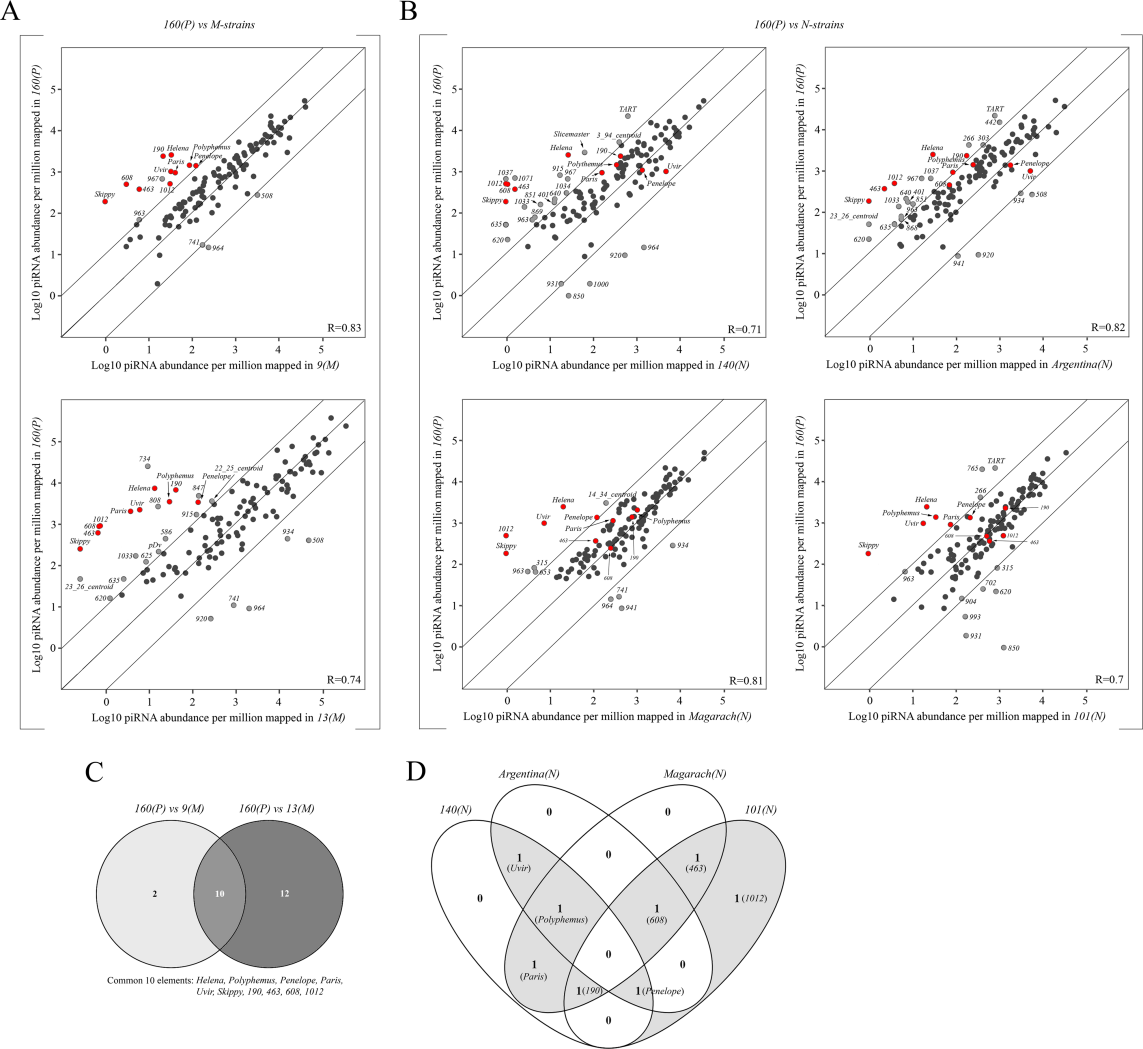
Comparative analysis of the ovarian piRNA profiles between *P*-like strain *160* and both *M*- and neutral *(N)* strains studied. A) and B) Scatter plots represent the result of pairwise comparison of normalized piRNAs (23–29 nt) in *P*-strain *160* versus *M*-like strains *9* and *13*, and in *P*-strain *160* versus *N*-strains *140*, *Argentina*, *Magarach* and *101*, respectively. Diagonal lines indicate 10-fold levels of difference. All the TEs that exceed 10-fold line are marked as gray dots. The red dots indicate TEs that are shared between *M*-strains *9* and *13* in terms of their low expression levels in comparison with *P*-strain *160*. Spearman’s correlation (R) is shown. C) Venn diagram depicting differences and similarities in a number of TEs exhibiting 10-fold lower piRNA expression level in *M*-strains *9* and *13* in comparison with *P*-strain *160*. 10 families show the same pattern of deficit in strains *9* and *13*, relative to strain 160. D) Venn diagram demonstrates distribution of piRNAs to eight essential elements distinguishing neutral strains from *M*-like in terms of piRNA-mediated silencing among studied *N*-strains.

This analysis indicates that the total repertoire of targets for small RNA silencing in strain *160(P)* is significantly higher than in all other studied strains (Figs 2A, 2B, S1A and S1B). Surprisingly, the global piRNA profile for known *D*. *virilis* TEs and other repeats is more similar between strain *160(P)* and *M*-strains (R(*160*:*9*) = 0.83; R(*160*:*13*) = 0.74, Spearman’s correlation coefficient) than between strain *160(P)* and several *N*-strains (R(*160*:*140*) = 0.71; R(*160*:*101*) = 0.7) (Fig 2A and 2B). This suggests the possibility that protection is not mediated by a general maternal piRNA profile, but rather to certain specific TEs yet to be identified. To identify such candidates, we compared sets of piRNA targets distinguishing strain *160(P)* from both typical *M*-strains, *9* and *13*, and obtained a list of ten TEs in common across comparisons (Fig 2C). These are TEs for which piRNAs are more abundant in strain *160(P*) when compared to both *M*-strains: *Polyphemus*, *Penelope*, *Paris*, *Helena*, *Uvir*, *Skippy*, *190*, *463*, *608*, and *1012*. However, comparing *160(P)* and *N*-strains, we find that piRNAs from *Helena* and *Skippy* are uniquely found at high levels in strain *160(P)*. Thus, if neutrality is conferred by piRNAs that uniformly target the same TE family or families, *Helena* and *Skippy* piRNAs are not likely to be required to prevent HD. However, among the eight remaining candidates, there is no shared family among the neutral strains (*N-*strains and *160(P))* that have a piRNA profile that is similar across strains. For example, in contrast to *160(P)*, *Penelope*-derived piRNAs are more lowly expressed in strain *Magarach(N)*, *Polyphemus*-targeted piRNAs are more lowly expressed in strain *101(N)* and, finally, *Paris*-related piRNAs are lowly expressed in strain *Argentina(N)* and in strain *101(N)* (Fig 2D). Thus, we failed to detect one candidate causative TE or combinations of certain TEs present in all neutral strains whose piRNAs guarantee immunity to HD (Fig 2D). This suggests the possibility that maternal protection in crosses with strain *160(P)* males may be conferred by different mechanisms across the strains.

A similar comparative analysis of siRNA expression between strain *160(P)* and *M*-strains demonstrated that siRNAs complementary to only *Penelope* and *Helena* elements are absent in the ovaries of strain *9(M)* and *13(M)* (S1A and S1B Fig). However, we detected *Penelope*-homologous siRNAs only in half of the studied neutral trains i.e. strains *Argentina* and *140* (S1C Fig).

In the context of immunity to HD syndrome manifestations, probably the most important condition is to constantly maintain effective piRNA production in the germline. It is well known that ovarian piRNA pools consist of molecules generated by primary and secondary processing mechanisms. Due to germline expression of Ago3 and Aub proteins necessary for secondary processing (“ping-pong” amplification), the germline specific piRNA pool can be assessed quantitatively by counting of “ping-pong” pairs [2,23]. We analyzed the “ping-pong” signature of piRNAs to the selected TEs and showed that these piRNA species contain ping-pong pairs in varying degrees (S2 Fig). Importantly, all of them exhibit a signature of secondary piRNA processing indicating that production of these piRNAs takes place in the germline but each element lacks such a ping-pong signature in at least one or more of the neutral strains. In addition, *Penelope* expression was previously shown to be germline-specific by whole-mount RNA in situ hybridization [24]. In the present study, using the same technique with the ovaries of *P*-strain *160*, we confirmed that *Paris*, *Polyphemus* and *Helena* elements exhibit germline-specific expression pattern as well (S3 Fig).

We further examined the pattern of divergence among piRNAs that map to the consensus TEs since piRNAs derived from divergent sequences are likely derived from degraded TE insertions. Among the selected HD-implicated TEs, the ovarian piRNA pool contains a very small amount of *Paris*-targeting piRNAs that were detected only in two studied *N*-strains—*140* and *Magarach*. Interestingly, only 10% of both sense and antisense-oriented piRNAs apparently originate from modern active copies of *Paris* elements while the rest of the *Paris*-complementary piRNAs were produced from ancestral highly diverged ones (S4 Fig). The same applies to the *Penelope*-derived piRNAs in strain *101(N)*. All other piRNA species to HD-implicated TEs, especially in the antisense-orientation, in all studied neutral strains were practically identical to the consensus and, hence, apparently originated from active copies of these elements (S4 Fig). This analysis further indicates that there is no active candidate inducer family, represented by sequence similar piRNAs, shared across all six neutral strains.

Overall, these data indicate that, in terms of piRNA-mediated protection to HD in *D*. *virilis* neutral strains, there is no general rule in the context of ovarian piRNAs complementary to particular TEs implicated in HD. In other words, in neutral strains the maternally transmitted piRNA pool may include different amounts of piRNAs corresponding to various TEs and the repertoire of these TEs often radically differs between the strains with same cytotype.

### Propagation and expression of *Penelope* does not change *M*-like cytotype

Syndromes of HD are explained by maternal protection against paternal induction and *Penelope* has long been considered the primary driver of paternal induction [18,20,22]. In the previous section we demonstrated that maternal piRNAs that target *Penelope* are not *necessary* to confer neutrality but, as neutrality may arise through different mechanisms, we sought to determine whether *Penelope* was either sufficient for induction or *Penelope* piRNA sufficient for protection. We thus characterized a simulation of natural invasion through the analysis of two transgenic strains of *D*. *virilis* containing full-size *Penelope* copies introduced into a typical *D*. *virilis M*-like strain *9* (the stock is assigned as *w3*) originally devoid of functional copies of this TE. Our previous experiments demonstrated that introduced *Penelope* underwent active amplification and occupied more than ten sites in the chromosomes of the transgenic strains [19]. However, at that time (in 2012) we did not detect any *Penelope*-derived small RNA species in these transgenic strains.

**Fig 3:**
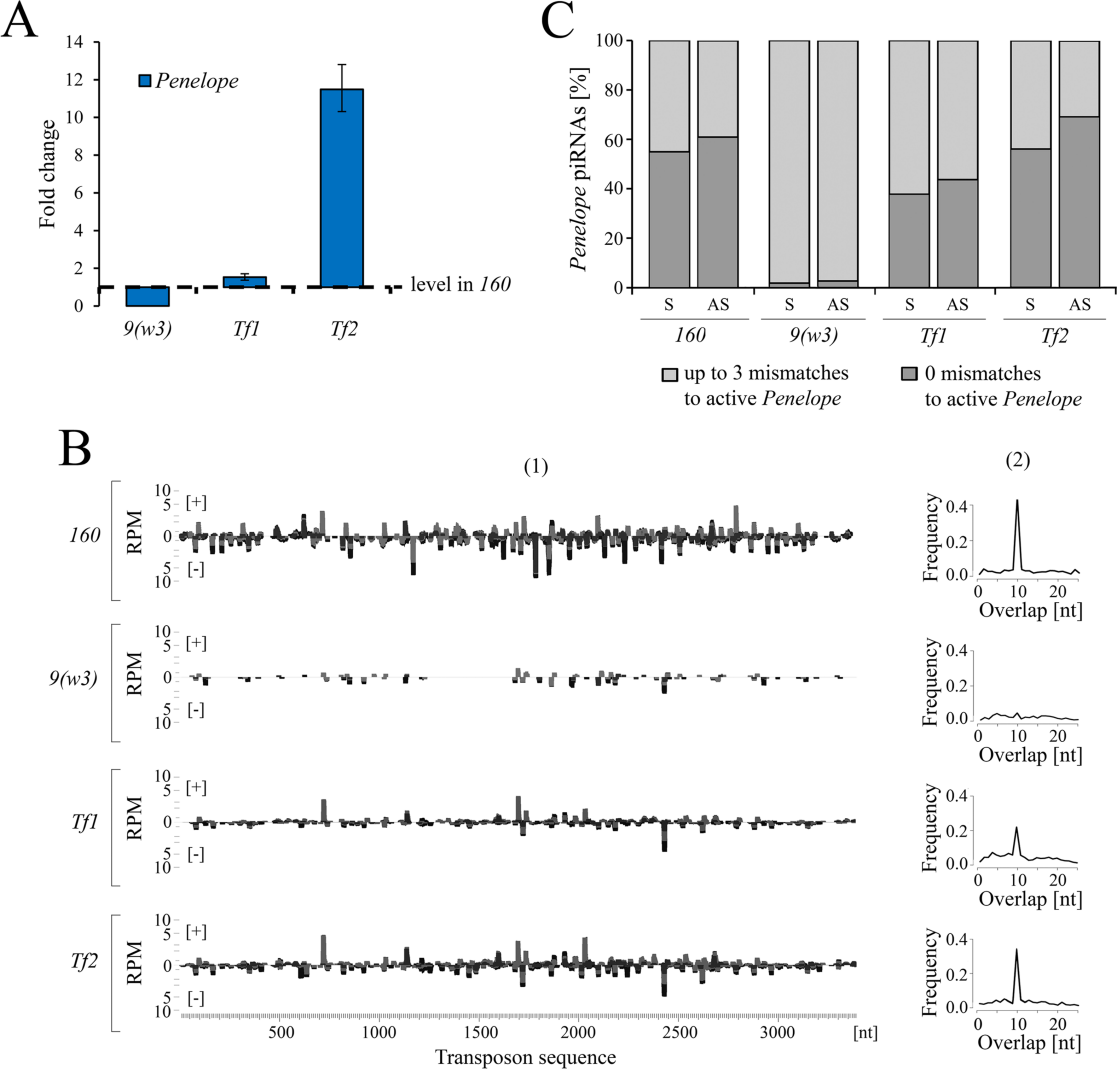
Characterization of *Penelope* activity in the ovaries of *Penelope*-transformed strains. A) Expression levels of *Penelope* in *9(w3)*, *Tf1*, *Tf2* strains relative to *P*-strain *160*. B) (1) The coverage of normalized *Penelope*-piRNA reads (23–29 nt) on the entire body of the element, across transformed strains. Sense reads are shown as [+], antisense as [–]. (2) The ping-pong signature of *Penelope*-derived piRNAs. C) Mapping proportions of *Penelope*-piRNAs to canonical sequence of the element with the perfect match and with the assumption of up to 3 mismatches.

Subsequent to the early analysis performed in 2011–2012, we have now found that *Penelope* is actively transcribed in these two strains and exhibits steady-state RNA levels equal to or even higher than in strain *160* (Fig 3A). We further observed piRNAs in both transgenic strains, indicating that some of *Penelope* copies acquired the properties of piRNA-generating locus (Fig 3B). Thus, in strain *Tf2* the level of piRNAs homologous to *Penelope* is only half as much as that observed in *P*-like strain *160*. The analysis of *Penelope*-derived piRNAs indicates a distribution of piRNAs along the entire *Penelope* body and clear-cut ping-pong signature (Fig 3B).

Similar to strain *160*, more than half of the *Penelope*-derived piRNAs in both strains originate from active and highly similar *Penelope* copies with few mismatches to the canonical sequence (Fig 3C). In contrast, *Penelope* piRNAs identified in the untransformed *M*-like strain *9(w3)* are highly divergent and likely derive from inactivated *Penelope* copies (termed “Omegas”) located in heterochromatic regions of the genome [25,26]. Interestingly, the pool of *Penelope* derived small RNAs in transgenic strains are primarily piRNAs. This is in contrast to inducer strain *160* and *D*. *melanogaster* strains transformed with *Penelope* [19], where *Penelope*-derived siRNAs are the major class (S5 Fig).

**Fig 4:**
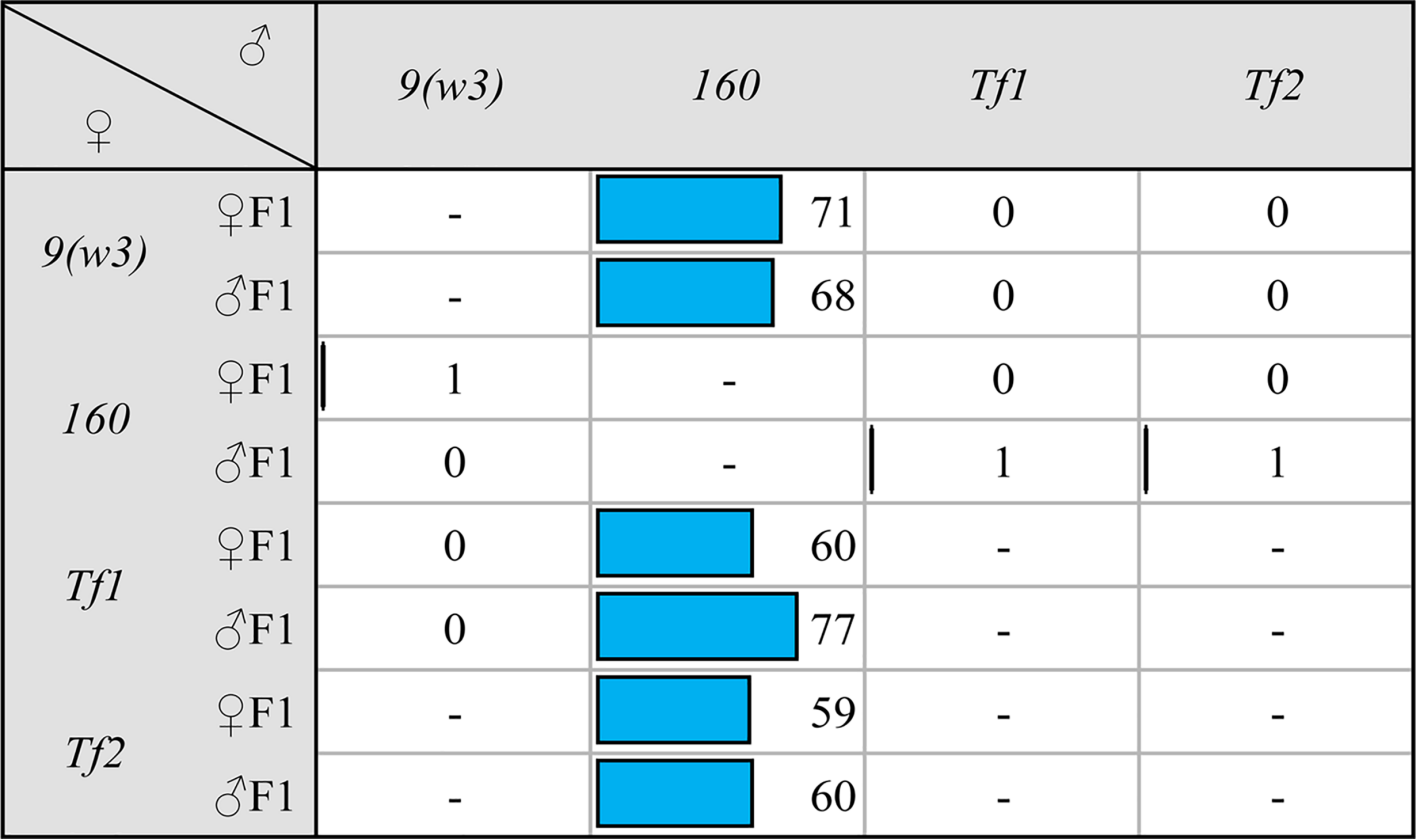
The frequency (in %) of gonadal atrophy in the progeny of dysgenic and reciprocal involving *P*-like strain *160* and *Penelope*-transformed strains. The number of examined individuals was ~100 separately for females and males.

Surprisingly, both transgenic strains containing multiple *Penelope* copies and abundant piRNAs behave exactly as the original *M*-like strain *9* in dysgenic crosses (Fig 4). They neither have the capacity to induce HD paternally nor protect against HD maternally. Therefore, the introduction of full-size *Penelope* into an *M*-like strain accompanied by its propagation, active transcription and piRNAs production was not sufficient to modify the cytotype. These results also indicate that the presence of piRNA complementary to *Penelope* in the oocyte is not the only prerequisite to prevent gonadal sterility when crossed with males of *P*-like strain *160*. Along these lines, it has been shown recently that the number of *P*-element and *hobo* copies *per se* has very little influence on gonadal sterility suggesting that HD is not determined solely by the dosage of HD-causative elements [27].

### Strain cytotype can be altered without amplification of TEs implicated in HD

The above results demonstrate that the maternal piRNAs that target all, or even most, asymmetric TEs that likely cause dysgenesis are not necessary to confer neutral strain status (Fig 2). Furthermore, *Penelope* piRNAs are not sufficient for maternal protection and the presence of active *Penelope* copies is not sufficient for paternal induction (Figs 3 and 4). This begs the question: What are the necessary and sufficient factors of HD in *D*. *virilis*? Among the analyzed strains, neutral strain *101* represents a special case. This is due to the fact that the genome of this strain does not produce piRNAs to the most-described HD-implicated TEs, e.g. *Paris*, *Helena*, *Polyphemus* and a very small amount of divergent *Penelope*-homologous piRNAs (Figs 2 and S4).

**Fig 5:**
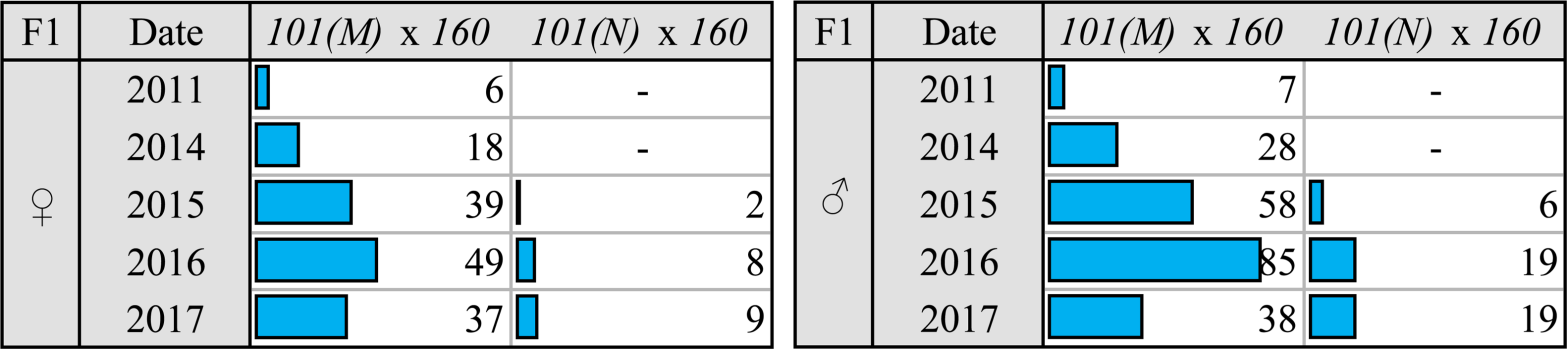
The alteration of the frequency (in %) of female and male gonadal atrophy in initially neutral strain *101*. The number of examined individuals was ~100 separately for females and males for each indicated time of monitoring.

In the course of our long-term monitoring of the gonadal atrophy observed in the progeny of dysgenic crosses involving *P*-like strain and various laboratory and geographical strains of *D*. *virilis*, we often observed significant variation in the level of sterility in the progeny of the same crosses occurring with time. Strikingly, among these strains, we have identified a spontaneous change from neutral cytotype to *M*-like one. Thus, while an old laboratory strain *101* kept in the Stock Center of Koltzov Institute of Developmental Biology RAS maintained a neutral cytotype for the whole period of observation (2011–2017) the same strain kept in our laboratory gradually became *M*-like strain (Fig 5).

**Fig 6:**
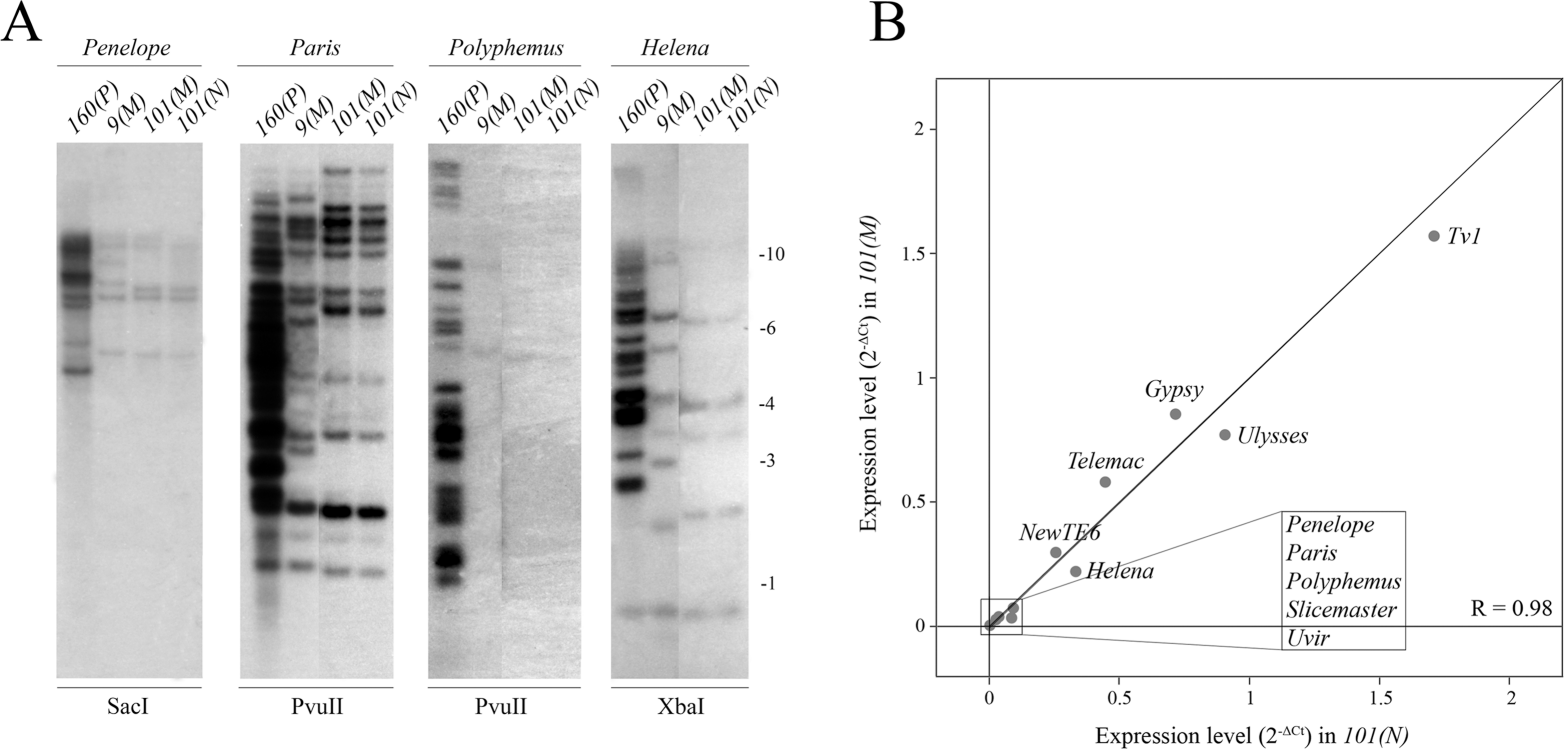
Genomic abundance and expression levels of putative HD-implicated TEs (*Penelope*, *Paris*, *Polyphemus* and *Helena*) in both cytotype variants of strain *101*. A) Southern blot analysis of genomic DNA of *160(P)*, *9(M)*, *101(M)* and *101(N)* strains. B) Expression levels of described *D*. *virilis* TEs in the ovaries of both variants of strain *101*. Spearman’s rank test was used to calculate the correlation (R) between the strains studied.

We considered the possibility that this shift in cytotype could be explained by changes in the TE profile between the strains. Surprisingly, Southern blot and PCR analyses demonstrate that *101 N-* and *M-* substrains have identical TE profiles for *Penelope*, *Paris*, *Polyphemus* and *Helena* (Figs 6A and S6). Additionally, qPCR analysis failed to detect any significant changes in the expression levels of the major asymmetric TEs as well as other described TEs in the compared variants (neutral *vs M*-like) of this strain (Fig 6B). These data rule out the possibility of strain contamination with a lab *M*-strain.

### Cytotype shift is accompanied by an altered piRNA and chromatin profile for a new set of repeats

**Fig 7:**
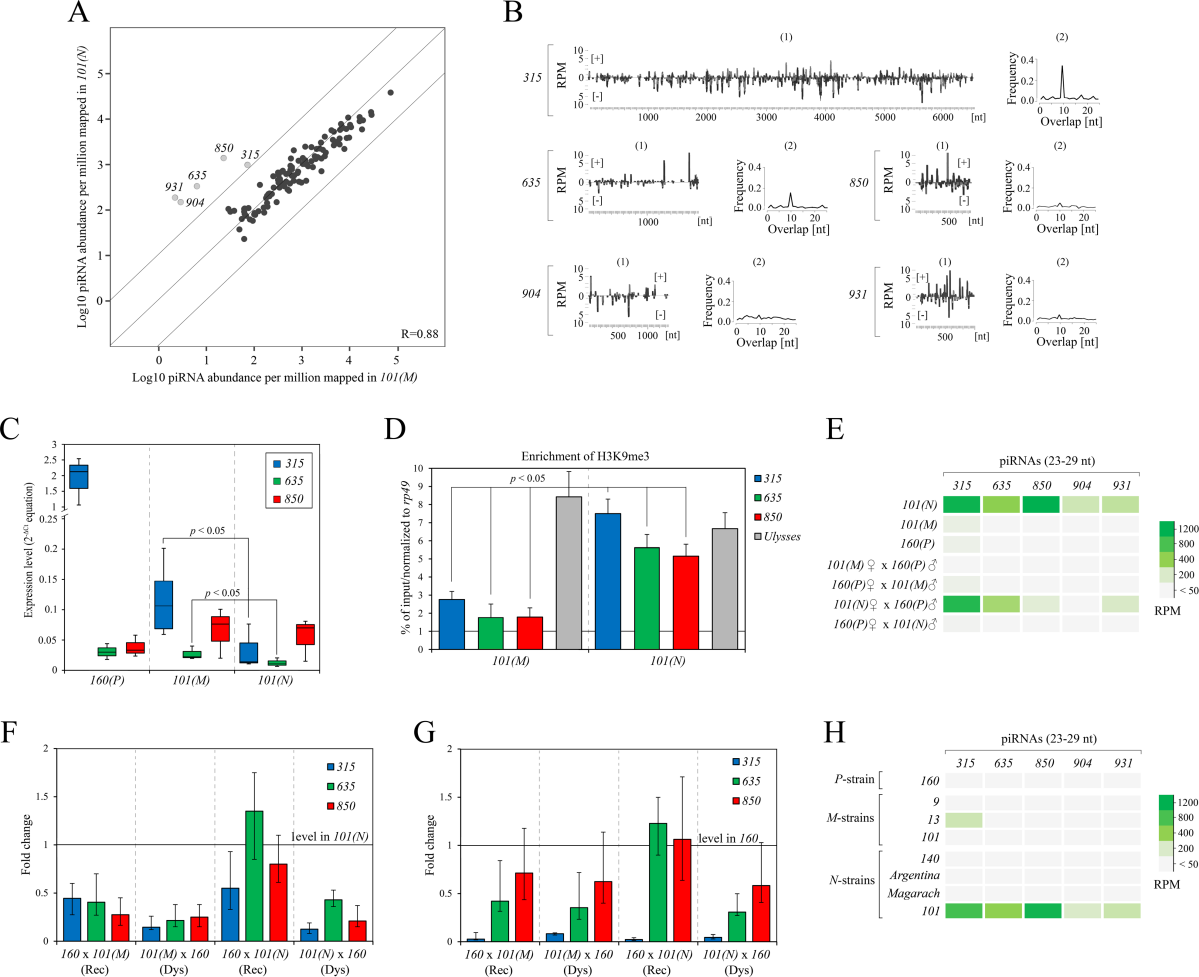
Characterization of small RNA mediated silencing in substrains of *101* exhibiting different cytotype. **A)** Scatter plot represent the result of pairwise comparison of normalized piRNAs (23–29 nt) in *M*-like strain *101* versus its neutral variant. Diagonal lines indicate 10-fold levels of difference. Gray dots indicate the repeats exhibiting more than 10-fold greater piRNA level. Spearman’s rank test was used for correlation (R) calculation. B) (1) The coverage of normalized *315*, *635*, *850*, *904* and *931*-derived piRNA reads (23–29 nt) from the *101(N)* strain on the entire body of correspondent elements. Sense reads are shown as [+], antisense as [–]. (2) The ping-pong signature of *315*, *635*, *850*, *904* and *931*-derived piRNAs. C) Expression levels of *315*, *635*, *850* elements in the ovaries of *160(P)*, *101(M)* and *101(N)* strains. *P*-values were calculated using *t*-test. D) Enrichment of H3K9me3 mark on the genomic loci carrying *315*, *635* and *850* using ChIP-qPCR on ovaries of substrains *101*. E) Heatmap of the ovarian expression of *315*, *635*, *850*, *904*, *931*-derived piRNAs in dysgenic and reciprocal hybrids. F) Expression levels of *315*, *635*, *850* elements in the progeny from dysgenic and reciprocal crosses relative to parental *101(N)* levels and G) to *160(P)* levels. H) Heatmap represents expression pattern of *315*, *635*, *850*, *904*, *931-*targeted piRNAs among the studied *P*-like, *M*-like and neutral strains.

To understand the observed differences in the cytotype of strain *101* variants we performed additional small-RNA sequencing. Indeed, the piRNA profile of strain *101(N)* has significantly higher piRNA levels (compared to *101(M))* for five previously undescribed repeats (*315*, *635*, *850*, *904* and *931*) (Fig 7A), indicating that differences in cytotype could be attributed to these repeats. Among these piRNA species, only piRNAs targeting *315* and *635* elements comprise many ping-pong pairs and, hence, are generated predominantly by germline-specific secondary processing mechanism (Fig 7B). Based on sequence similarity to the TE consensus, at least 25% of antisense-oriented piRNA molecules apparently originated from modern active elements, with the exception of piRNAs targeting the *904*-element (S7A Fig).

Focusing on the three elements (*315*, *635*, *850*) with maximal piRNA expression levels, we compared both variants of strain *101* in more detail to determine if differences in repeat profile could explain differences in cytotype. Element *315* encodes three open reading frames (ORF). According to the protein-domain structure, two ORFs appear to encode *gag* and *pol* genes. The third ORF has no homology to the described TEs and possibly encodes an *env* gene. Thus, element *315* probably represents a retroelement. Since we failed to find any homology of the *315* element to the described families of TEs in *Sophophora* subgenus we propose that this element is an exclusive resident of *Drosophila* subgenus. Element *635* has some homology to the *Invader* element of *D*. *melanogaster*, which belongs to the *Gypsy* family of LTR-containing retrotransposons. However, it has no long terminal repeats (LTRs) in its sequence. Finally, short *850* element (749 nt) doesn’t encode any ORF and seems to be non-autonomous.

Importantly, based on Southern blot and PCR analysis, these particular repeats did not undergo amplification in the neutral variant of strain *101* and both compared substrains exhibit identical restriction patterns of these elements, similar to that of *P*-like strain *160* (S7B and S7C Fig). Hence, the observed cytotype shift as well as the differences in piRNA pool to these elements apparently do not stem from differences in copy number among *101* substrains. Interestingly, we observed a significant increase of expression levels of *315* and *635* elements (*p* < 0.05; *t*-test), but not *850*, in the ovarian mRNA pool of *M*-like substrain *101* compared to the neutral substrain (Fig 7C). Overall, these results demonstrate that the capacity for these repeats to produce piRNAs is lower in the *101(M)* strain, even in the absence of movement.

What could lead to differences in the piRNA profile for these repeats between the *101(N)* and *101(M)* strains in the absence of movement? Studies of piRNA-generating loci in *Drosophila* revealed that the H3K9me3 mark, which serves as a binding site to recruit HP1a and its germline homolog Rhino, is required for transcription of dual-strand piRNA-clusters and transposon silencing in ovaries [2,28,29]. We hypothesized that a shift of the chromatin state in strain *101* modified the ability of particular genomic loci, carrying *315*, *635*, *850* elements, to produce piRNA species. These changes in piRNA profile may be an indication of a chromatin-based modification that may confer resistance to HD sterility in the neutral *101* substrain. To test this hypothesis, we estimated the levels of H3K9me3 and HP1a marks by ChIP combined with qPCR analysis in the ovaries of two cytotype variants of strain *101*. The analysis showed significant increase of H3K9me3 levels on genomic regions containing *315*, *635* and *850* elements (enrichment > 2.5, *p* < 0.05) as well as slight increase of HP1a enrichment in the neutral variant of strain *101* compared to the *M*-like substrain (Figs 7D and S8). In turn, *Ulysses* carrying regions used as a control demonstrated equal levels of the H3K9me3 mark, consistent with *Ulysses*-targeting piRNA levels being almost equal in the strain *101* variants (Fig 7D). This indicates that certain repeats have experienced shift in their chromatin profile, but that this shift is not global. A similar phenomenon has been recently described in *I-R* HD system in *D*. *melanogaster* [30]. In that comparative analysis of two reactive strains (weak and strong), it was shown that despite having a similar number of copies of the *I*-element, these strains significantly differ by enrichment of Rhino at the *42AB* piRNA-cluster containing *I*-elements remnants. Furthermore, a lower level of *I*-element targeted piRNA species was observed in the strong-reactive strain as a result [30].

Given these differences, it is possible that these elements are the primary drivers of dysgenesis in *D*. *virilis*. To further test the hypothesis that activation of these elements could contribute to HD, we compared first piRNA levels of all these elements in the ovaries of the F1 progeny from dysgenic-like and reciprocal crosses using variants of strain *101* and *P*-like strain *160*. These experiments demonstrate that piRNAs targeting *315*, *635*, and *934* elements showed similar levels in the ovaries of F1 hybrids from dysgenic crosses (*101(N)* x *160*) and parental neutral strain *101*, but lower levels in progeny of reciprocal crosses where such piRNAs would not be maternal (*160* x *101(N)*) (Fig 7E). Thus, the maternally provisioned piRNAs complementary to *315*, *635* and *931* elements are required to stimulate the generation of the corresponding piRNAs in the progeny, as shown in other systems of HD [3,4]. However, in the analysis of steady-state mRNA levels of these TEs in the ovaries of dysgenic and reciprocal progeny of crosses between *101* substrains and *P*-like strain *160*, we failed to obtain any induction of *315*, *635* and *850* elements exceeding their levels of parental strains (Fig 7F and 7G). On the contrary, the ovaries of F1 hybrids from the reciprocal (non-dysgenic) crosses involving strains *101(N)* males and *160(P)* females showed even significantly higher expression levels of these elements in comparison to dysgenic ones. Moreover, the dysgenic and reciprocal hybrids of *M*-like substrain *101* and strain *160(P)* showed no differences in the mRNA levels of the studied elements (Fig 7F and 7G). These results indicate that activation of these elements *per se* is unlikely to be causative to HD because *101(N)* and *101(M)* have identical TE profiles. We therefore considered the possibility that what distinguishes strain *101(N)* from *101(M)* may have an epigenetic basis or, alternately, an unknown genetic change that alters repeat chromatin. If so, then lack of piRNAs to these elements in *101(M)* could explain the *M-*cytotype. To test this, we compared piRNA levels and family level abundance with inducer strain *160(P)*. Critically, none of these elements show increased piRNA levels in strain *160(P)* compared to strain *9(M)* (Fig 7H). Thus, asymmetry in the piRNA pool for these particular elements is not a necessary condition for dysgenesis.

According to the recent studies differences in parental expression levels of genic piRNAs may contribute to the dysgenic manifestations in the progeny [18,30]. With this in mind, we compared the expression of genic piRNAs in the ovaries of both *101* substrains and did not observe significant differences in their levels (S9A Fig). Ping-pong of genic piRNA profiles are also exhibit high similarity between these strains (S9B Fig). Based on these data, we concluded that differences in genic piRNAs unlikely have impact on the observed cytotype shift.

Overall, we have shown that the enrichment of heterochromatic marks (H3K9me3 and HP1a) in the genomic regions containing *315*, *635* and *850* elements is significantly lower in *M*-like variant of strain *101* compared to neutral one. Together, these data provide further evidence that the mechanism of maternal repression may significantly vary among strains. However, additional experiments involving Rhino ChIP and genome sequencing of strain *101* are needed to clearly prove this assumption and identify the loci responsible for the enhanced piRNA production in one of the two *101* substrains.

### Ovarian levels of mRNA and complementary piRNAs for many asymmetric TEs are similar in dysgenic and reciprocal hybrids of *D*. *virilis*

One of the main consequences of activation of a particular asymmetric TE in the progeny of dysgenic crosses is their expression level excess compared to both paternal strains and reciprocal hybrids [3,15,18,31]. Studies of the *I-R* syndrome of HD in *D*. *melanogaster* demonstrate higher expression of the *I*-element in the F1 progeny from dysgenic crosses compared to reciprocal ones [3,30,31]. This is due to the maternal deposition of piRNAs targeting the *I*-element and its effective silencing in only one direction of the cross. Additionally, various studies of HD systems, including the *D*. *virilis* syndrome, demonstrated that transgenerational inheritance of piRNAs is able to trigger piRNA expression in the next generation by changing the chromatin of piRNA-clusters due to paramutation [3,4,32–34]. However, a pattern of higher TE expression in the absence of complementary maternal piRNA is less apparent in *D*. *virilis*. Despite strain asymmetry in genomic content and piRNA abundance of *Penelope* and several other TEs, germline piRNA pools do not differ drastically between reciprocal F1 progeny, with the exception of *Helena* element [18]. We therefore sought to determine whether this atypical pattern was also observed in crosses with other strains, focusing on asymmetric *Penelope*, *Paris*, *Polyphemus* and *Helena* as well as *Ulysses* present in all strains.

**Fig 8:**
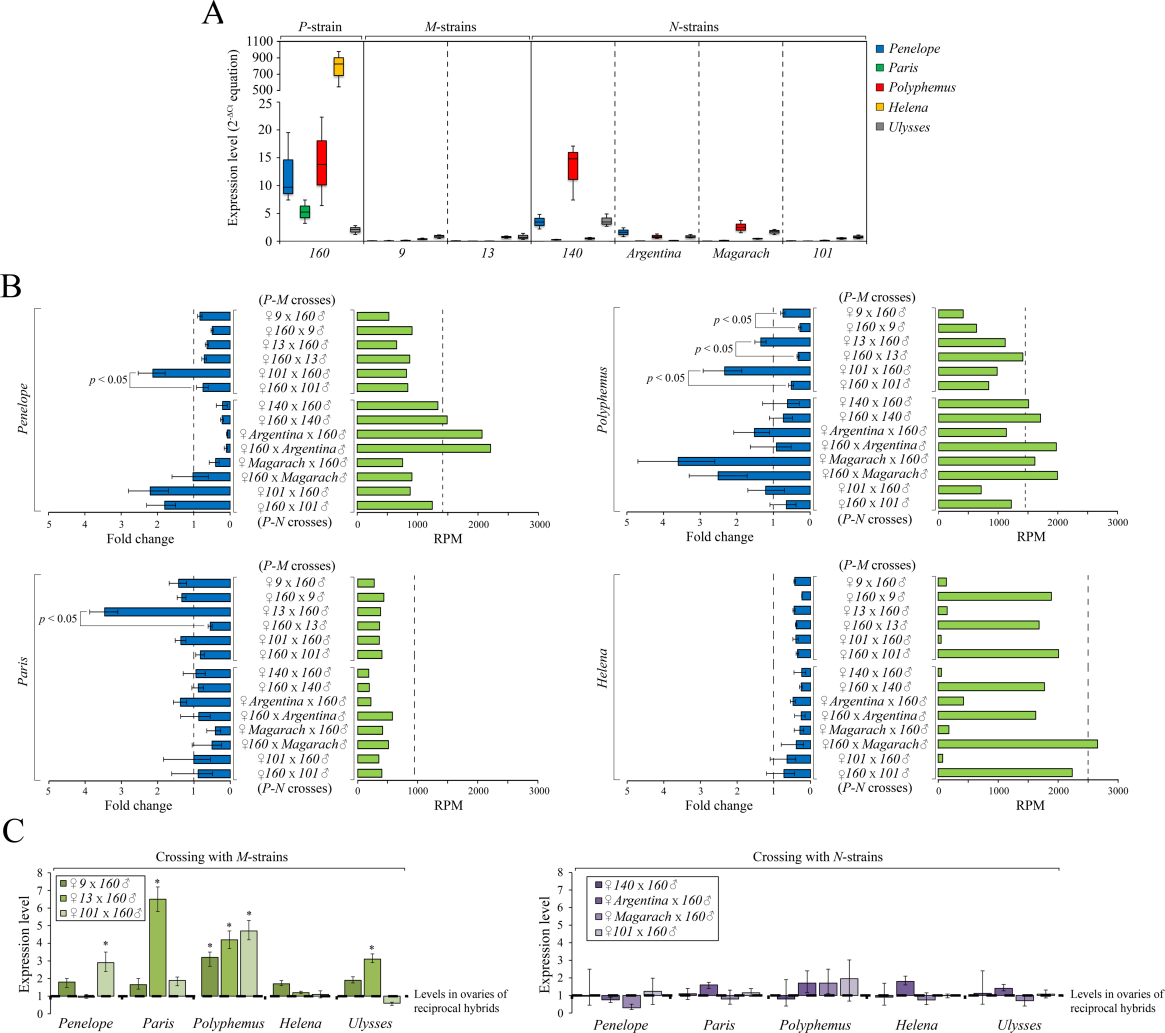
Comparative analysis of a set of HD-implicated TEs in the ovaries of dysgenic and reciprocal hybrids. A) Expression levels of *Penelope*, *Paris*, *Polyphemus*, *Helena* and *Ulysses* among the studied *P*-like, *M*-like and neutral strain. B) mRNA and piRNAs expression levels in the ovaries of the progeny from dysgenic and reciprocal hybrids. At the left—expression levels of indicated TEs relative to the level in *P*-strain *160*. At the right–normalized piRNAs expression levels. The dotted line indicates level in *P*-strain *160*. *P*-values were calculated using *t*-test. C) Expression level of *Penelope*, *Paris*, *Polyphemus*, *Helena* and *Ulysses* in the ovaries of dysgenic crosses compared to reciprocal ones. *P*-values were calculated using *t*-test.

As expected, ovarian mRNA levels revealed a complete correspondence with the piRNA expression levels among strains (Figs 8A, 2A and 2B). For example, we detected both *Penelope* mRNA and piRNA expression in *140(N)* and *Argentina(N)*, but neither were evident in *Magarach(N)* and *101(N)*. However, in all cases when females from *M*-like strains are crossed with strain *160* males, ovarian levels of expression are uniformly significantly higher for only one asymmetric TE–*Polyphemus* (fold change 3, 5, 3.5, *p* < 0.05, *t*-test, in dysgenic hybrids with strains *9(M)*, *13(M)* and *101(M)*, respectively) (Fig 8B and 8C). In most cases the observed differences in expression for *Penelope* and *Paris* elements in the ovaries of dysgenic and reciprocal hybrids were not dramatic and when exist rarely exceed 1.5–2 fold. Moreover, in the crosses involving neutral strains and strain *160*, we failed to detect any characteristic differences in TEs expression between reciprocal hybrids (Fig 8B and 8C). Thus, independent of maternal piRNA profile, all reciprocal crosses with neutral strains show similar levels of expression. However, the two variants of strain *101* give different results when crossed with *P*-like strain *160*. In spite of the fact that *101* substrains contain equal levels of piRNAs complementary to the HD-implicated TEs, in the case of the *M*-like variant we observed higher levels of expression in the dysgenic hybrids for *Penelope* (fold change 3; *p* < 0.05, *t*-test) and *Polyphemus* (fold change 3.5, *p* < 0.05, *t*-test). Moreover, increase of *Ulysses* element (found in all *D*. *virilis* strains) expression (fold change 3, *p* < 0.05, *t*-test) was demonstrated in the dysgenic ovaries of *13(M)* and *160(P)* hybrids (S10A Fig). These results demonstrate that factors other than maternal piRNA abundance lead to variation in resident TE expression in crosses between strain *160* and *101* substrains. For the neutral *101* strain, we failed to detect significant differences in the hybrids from both directions of crosses for any of TEs tested (Fig 8B).

With the exception of a few TEs and repeats, piRNA abundance in the ovaries from dysgenic and reciprocal progeny exhibited no drastic differences including piRNAs complementary to asymmetric TEs (Figs 8B and S11). Surprisingly, *Helena*, which maintains high level of asymmetry of the maternal pool of piRNAs in the progeny, exhibits very similar levels of correspondent mRNA expression in the hybrids obtained in both directions of crosses (Fig 8B). In spite of overall similarity, piRNA pools in the ovaries of F1 progeny are able to comprise significantly different number of ping-pong pairs to all of transposons studied (S10B Fig). For example, in the ovaries from dysgenic progeny (strain *160* males) with strains *9(M)* and *Argentina(N)* females, the number of ping-pong pairs to *Penelope*, *Paris* and *Polyphemus* was 2-3-fold lower than in the ovaries from reciprocal hybrids (S10B Fig). We have also found that enrichment of the H3K9me3 mark on *Penelope*, *Paris*, *Polyphemus* and *Helena* sequences does not differ significantly in the F1 progeny of dysgenic and reciprocal crosses (S10C Fig). Thus, we propose that piRNA-mediated transcriptional gene silencing of these HD-implicated TEs is similar in both directions of crosses and maternally provisioned piRNAs to these TEs are not necessary to stimulate the production of correspondent piRNA species in the progeny. These results are in agreement with recently published data [18].

In summary, it should be emphasized that in contrast to the *I-R* system in *D*. *melanogaster*, where maternal deposition of *I*-element piRNAs results in dramatic increase of piRNA expression targeting *I*-element in the progeny and efficient suppression of *I*-element activity, in *D*. *virilis* maternally provisioned piRNAs do not always guarantee efficient generation of the correspondent piRNAs in the progeny to maintain silencing of complementary TEs and provide adaptive genome defense. We conclude that in *D*. *virilis* the determination of asymmetric TEs expression levels in the ovaries of the progeny from dysgenic and reciprocal crosses does not allow one to unambiguously assign causality for HD to specific TE families. This fact points to an alternate mode of HD in *D*. *virilis*.

## Conclusions

The standard explanation for the phenomenon of hybrid dysgenesis is that TEs inherited paternally become germline activated in the absence of maternal piRNA. Here, however, we suggest that repression of paternal TEs by maternal piRNA may not be the sole mechanism of protection against this form of hybrid sterility. Using the *D*. *virilis* system of HD as a model, we have demonstrated that neutral strains exhibiting “immunity” to hybrid dysgenesis in *D*. *virilis* do not share a consistent pattern of piRNAs complementary to TEs likely causative of dysgenesis. Strikingly, the introduction and propagation of one presumably causal TE (*Penelope*) in the genome of *M*-like strain does not even change the cytotype of the transformed strains. Finally, we identified a shift of cytotype from *N* to *M* that occurred in a strain without changes in the expression or copy number of asymmetric TEs implicated in HD. The observed “immunity” of neutral strains to HD manifestations is apparently established by an increased repertoire of repeats that become targets for piRNA biogenesis as well as a modified chromatin state of several genomic regions compared to *M*-like strains. These studies suggest that hybrid dysgenesis in *D*. *virilis* cannot be solely explained by the well-established “hybrid dysgenesis paradigm”, developed in *D*. *melanogaster*. Rather, other properties of the genome contribute to maternal protection. The precise molecular mechanisms underlying “susceptibility” of *D*. *virilis* strains to TEs invasion requires further investigation.

## Materials and methods

### Fly strains and crosses

Seven *D*. *virilis* strains, namely *9* (Batumi, Georgia), *13* (Krasnodar, Russia), *101* (Japan), *Argentina* (Argentina), *Magarach* (Crimea, Russia), *140* (laboratory strain) and laboratory strain *160* were used in this study. Original fly stocks are available in the Stock Center of Koltzov Insitute of Developmental Biology RAS. In addition, two previously described *165* and *247* strains [19] were used, designated in this paper as *Tf1* and *Tf2*, respectively. These strains were obtained as a result of transgenesis of the full copy of *Penelope* retroelement into *9* strain (19). All flies were reared on standard agar-yeast-sugar-raisins medium at the constant temperature regime (25°C). Since the start of monitoring for cytotype shift (2011) stocks of both substrains of *101* (from the Stock Center of Koltzov Institute of Developmental Biology RAS and ours) are reared on the same medium and an equivalent population size (not more than 20 flies per vial) has been maintained.

The dysgenic crosses involved males of *P*-like *160* strain and females of all aforementioned strains (*9*, *13*, *101*, *Argentina*, *Magarach* and *140*). As a control, reciprocal crosses were performed. Monitoring and counting of gonadal atrophy were conducted as described [11].

### Small RNA libraries sequencing and bioinformatic analysis

Total RNA was extracted from ovaries of 7–10 days old females using Extract RNA reagent (Evrogen, Russia). Total RNA was extracted from both *101* substrains after the second confirmation of cytotype shift in 2016. To prepare the small RNA fraction for cloning, total RNA from ovaries (~ 25 μg) was separated, using 15% polyacrylamide gel electrophoresis containing 8 M Urea. After incubation in an ethidium bromide solution (0.5 μg/ml), gel fragments corresponding to the small RNA fraction were excised, using chemically synthesized RNA corresponding to 21 and 29 nts as size markers. Cloning of small RNA libraries was performed by Illumina TruSeq Small RNA prep kit (Illumina, USA) according to the manufacturer’s protocol. Sequencing was conducted on an Illumina NextSeq 500 platform.

As a result of deep-sequencing we obtained 5–14 million reads of small RNAs for each library. Pre-processing procedure included: 3’-adapter trimming, filtration of reads by length (>18 nt) and quality (80% of nt have ≥ 20 Phred quality). Pre-processed reads were further subjected to subtraction of reads matching to all rRNA, tRNA, snRNA and miRNA sequences. The selected reads were mapped to the latest release of *D*. *virilis* genome by Bowtie [35], requiring a perfect match. In order to identify siRNAs and piRNAs, the sequenced reads were mapped to the canonical sequences of TEs obtained from combined libraries of annotated and computationally predicted *D*. *virilis* transposons and repeats sequences [18]. In addition, recently described *DAIBAM MITE* (GenBank: EU280326) and *Tetris* (GenBank: KF723713.1; KF723710.1) elements sequences were considered [36,37]. For genic piRNAs mapping the latest annotation of *D*. *virilis* transcripts was used (r1.06) and the alignment performed with requirement of perfect match. Less than 25 CPM of mapped siRNAs and piRNAs per transposons or transcripts were considered as lowly expressed and discarded. Length distribution and counting of siRNAs and piRNAs reads, nucleotide biases, ping-pong signatures, coverage of transposon sequence by piRNAs were analyzed with accordance to the well-described technique [23,38] using custom scripts written in Python. Venn diagrams were made using Venny 2.1 (http://bioinfogp.cnb.csic.es/tools/venny/). Scatter plots were created using Plotly (https://plot.ly).

### Quantitative PCR

For analysis, cDNA was prepared from 2 μg Turbo-DNAase (Ambion, USA) treated total RNA using oligo(dT) primer and MMLV reverse transcriptase (Evrogen, Russia). PCR was performed on ABI PRISM 7500 System (Applied Biosystems, USA). Detection of amplification products was carried out using SYBR Green 1 with the presence of ROX reference dye (Evrogen, Russia) in accordance with the manufacturer’s protocol. Quantification was normalized to ubiquitously expressed *rp49* gene and calculation of relative expression levels was performed using the equation 2^-ddCt^. Specificity of amplified products was verified by sequencing as well as melting curve analysis. The resulting value of the expression level for each sample was determined basing on at least three biological replicates. Sequences of used primers are shown in S1 Table.

### Whole mount *in situ* RNA hybridization

Dissection of ovaries, fixation, Proteinase K treatment, re-fixation and hybridization steps were performed as described [24].

Matrices for probe preparation were prepared by PCR (sequences of used primers are shown in S1 Table) of genomic DNA of *160* strain. Labeling of RNA probes with DIG-11-UTP (Roche, France) was made by MAXIscript T7 kit (Ambion, USA). Anti-DIG-AP antibodies (Roche, France) were used in 1:2000 dilution. Images obtained by binocular microscope Nikon Alphaphot-2 YS2 (Japan).

### Southern blotting

To analyze genomic TE profiles, DNA samples (~ 10 μg) were digested by restriction enzymes (*Penelope*–SacI; *Paris*, *Polyphemus* and *850* –PvuII; *Helena*–XbaI; *315* –NcoI; *635* –PvuI), fractionated through 0.8% agarose gel and transferred onto Hybond-XL membrane (Amersham Biosciences, USA). Hybridization was carried out at 60°C overnight in solution containing 6xSSC, 10 mM EDTA, 0.5% SDS and 5x Denhardt’s solution. The PCR-prepared matrices were used for probe preparation. Primers can be found in S1 Table. Labeling of probe with (P32)-dATP was performed using Decalabel kit (Thermo Scientific, USA).

### Semiquantitative PCR of genomic DNA

PCR was performed, in addition to Southern analysis, to compare the presence of full copies of TEs in the genomes of *160*, *9*, *101(N)* and *101(M)* strains. Amplification was performed using ScreenMix kit (Evrogen, Russia). PCR products were separated in 1.5% agarose gel including ethidium bromide (0.5 μg/ml) for detection. Reaction using primers to *rp49* gene represents a loading control. Used primers were the same as for probe preparation in previously described hybridization procedure.

### Chromatin immunoprecipitation and quantitative PCR analysis

Chromatin from *Drosophila* ovaries (~ 100 pairs for each IP experiment) was extracted and immunoprecipitated according to the published protocol [39]. ChIP experiments were carried out using commercially available antibodies anti-H3K9me3 (ab8898) (Abcam, UK) and anti-HP1a (C1A9) (DSHB). To bind antibodies Pierce protein A/G agarose (Thermo Fisher Scientific, USA) was used. Quantitative PCR was applied to evaluate the protein enrichment in the genomic loci. Percent of precipitated chromatin was calculated according to input values following normalization to actively transcribed *rp49* gene. The resulting value represents the mean of two biological replicates. The error is indicated by the standard error of mean (SEM). Primer sequences are presented in S1 Table.

### Statistical analysis

Student’s *t*-test was used to compare groups with each other (qPCR and ChIP-qPCR data). *P-values* ≤ 0.05 were considered statistically significant.

## Supporting information

S1 Fig Comparative analysis of the ovarian siRNA expression between *P*-like strain *160* and both *M*- and neutral (*N*) strains studied. A) and B) Scatter plots represent the result of pairwise comparison of normalized siRNAs (21 nt) in *P*-strain *160* versus *M*-like strains *9* and *13*, and in *P*-strain *160* versus *N*-strains *140*, *Argentina*, *Magarach* and *101*, respectively. Diagonal lines indicate 10-fold levels of difference. Gray dots indicate the TEs exhibiting more than 10-fold greater level of siRNAs between the pair of comparison. The results of Spearman’s correlation tests (R) are demonstrated. C) Venn diagram depicts differences and similarities in a number of TEs exhibiting 10-fold greater siRNA expression level in *M*-strains *9* and *13* in comparison with *P*-strain *160*.
(TIF)

S2 Fig The signature of secondary processing (ping-pong amplification) of piRNAs targeting selected ten significant TEs in *P*-strain *160* and studied neutral strains. *M*-strains were not plotted due to a very small expression of piRNAs and inability to calculate ping-pong signal.
(TIF)

S3 Fig Whole-mount *in situ* RNA hybridization of sense transcripts of *Polyphemus, Paris and Helena* and *Paris* in the ovaries of *D. virilis* strain *160*. (TIF)

S4 Fig Matching of piRNAs to canonical sequence of selected ten essential TEs in *P*-strain *160* and studied neutral strains. Number of mismatches (up to 3) to active element are indicated; piRNAs are split into sense (S) and antisense (AS) species.
(TIF)

S5 Fig Relative quantities of normalized siRNA (21 nt) and piRNA (23–29 nt) fractions homologous to *Penelope* in the ovarian pool of indicated strains. (TIF)

S6 Fig Semiquantitative PCR of *Penelope, Paris, Polyphemus* and *Helena* of genomic DNA of *160(P)*, *9(M)*, *101(M)* and *101(N)* strains. Primers correspond to the middle region of indicated TEs. *Rp49* gene serves as a loading control.
(TIF)

S7 Fig Matching of piRNAs and genomic abundance of *315*, *635* and *850* elements in substrains of *101*. A) Mapping of *315*, *635*, *850*, *904* and *931*-piRNAs to canonical sequence of the element in strain 101(N). B) Southern-blot analysis of genomic DNA of *160(P)*, *101(M)* and *101(N)* strains. C) Semiquantitative PCR of *315*, *635* and *850* elements of genomic DNA of *160(P)*, *101(M)* and *101(N)* strains. Primers correspond to the middle region of indicated TEs. *Rp49* gene serves as a loading control.
(TIF)

S8 Fig Enrichment of HP1a on the genomic loci carrying *315*, *635* and *850* using ChIP-qPCR on ovaries of substrains *101*. (TIF)

S9 Fig Genic piRNAs analysis in the substrains of *101*. **A)** Scatter plot represents the result of comparison of normalized piRNAs (23–29 nt) in *101M* and *101N* strains. Diagonal lines indicate 10-fold levels of difference. The results of Spearman’s correlation tests (R) are demonstrated. B) At the left–number of consitituted ping-pong pairs; at the right–frequency of overlapped piRNAs.
(TIF)

S10 Fig Comparative analysis of *Ulysses* and secondary piRNAs in the ovaries of dysgenic and reciprocal hybrids. A) mRNA and piRNAs expression levels in the ovaries of the progeny from dysgenic and reciprocal hybrids. At the left—expression levels of indicated TEs relative to the level in *P*-strain *160*. At the right–normalized piRNAs expression levels. The dotted line indicates level in *P*-strain *160*. P-values were calculated using *t*-test. B) The ratio of constituted ping-pong pairs between the dysgenic and reciprocal hybrids. C) H3K9me3 ChIP-qPCR on ovaries of dysgenic and reciprocal hybrids involving *P*-strain *160* and *M*-like strain *9*.
(TIF)

S11 Fig Comparative analysis of the ovarian piRNA profiles between dysgenic and reciprocal crosses involving strain *160* and both *M*- and neutral *(N)* strains. A) and B) Scatter plots represent the result of pairwise comparison of normalized piRNAs (23–29 nt) between dysgenic and reciprocal ovaries of strain *160* and *M*-strains and *160* and *N*-strains, respectively. Diagonal lines indicate 10-fold levels of difference. All the TEs that exceed 10-fold line are marked as gray dots. The results of Spearman’s correlation tests (R) are shown.
(TIF)

S1 Table Primers used in the study. (XLSX)
