# Supplementary figures and images for: Spontaneous gain of susceptibility suggests a novel mechanism of resistance to hybrid dysgenesis in Drosophila virilis
Source: PLoS Genet. 2018 May 29;14(5):e1007400. doi: 10.1371/journal.pgen.1007400 (PMC5993320; doi:10.1371/journal.pgen.1007400)

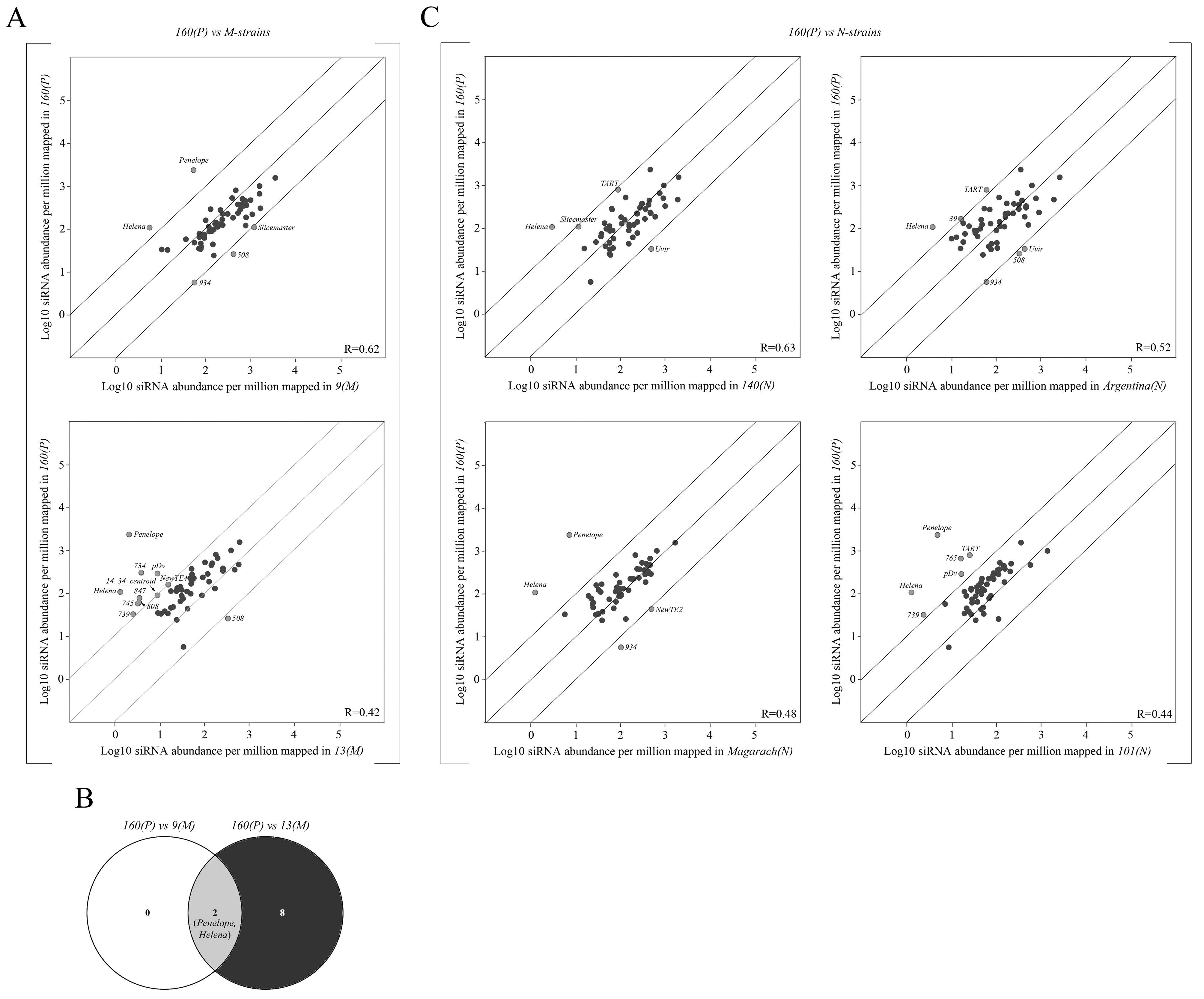

Supplement: S1 Fig — A) and B) Scatter plots represent the result of pairwise comparison of normalized siRNAs (21 nt) in P-strain 160 versus M-like strains 9 and 13, and in P-strain 160 versus N-strains 140, Argentina, Magarach and 101, respectively. Diagonal lines indicate 10-fold levels of difference. Gray dots indicate the TEs exhibiting more than 10-fold greater level of siRNAs between the pair of comparison. The results of Spearman’s correlation tests (R) are demonstrated. C) Venn diagram depicts differences and similarities in a number of TEs exhibiting 10-fold greater siRNA expression level in M-strains 9 and 13 in comparison with P-strain 160. (TIF) [file pgen.1007400.s001.tif]

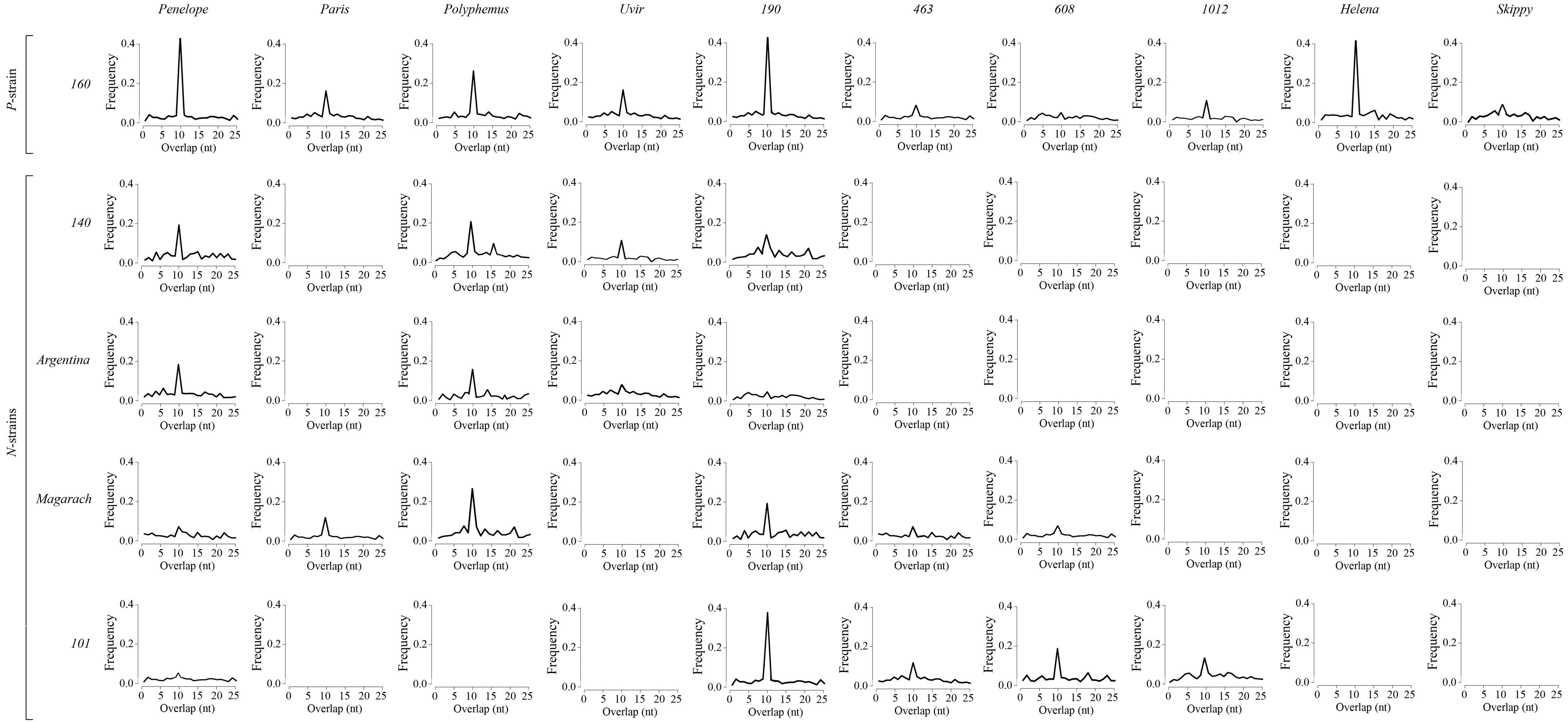

Supplement: S2 Fig — M-strains were not plotted due to a very small expression of piRNAs and inability to calculate ping-pong signal. (TIF) [file pgen.1007400.s002.tif]

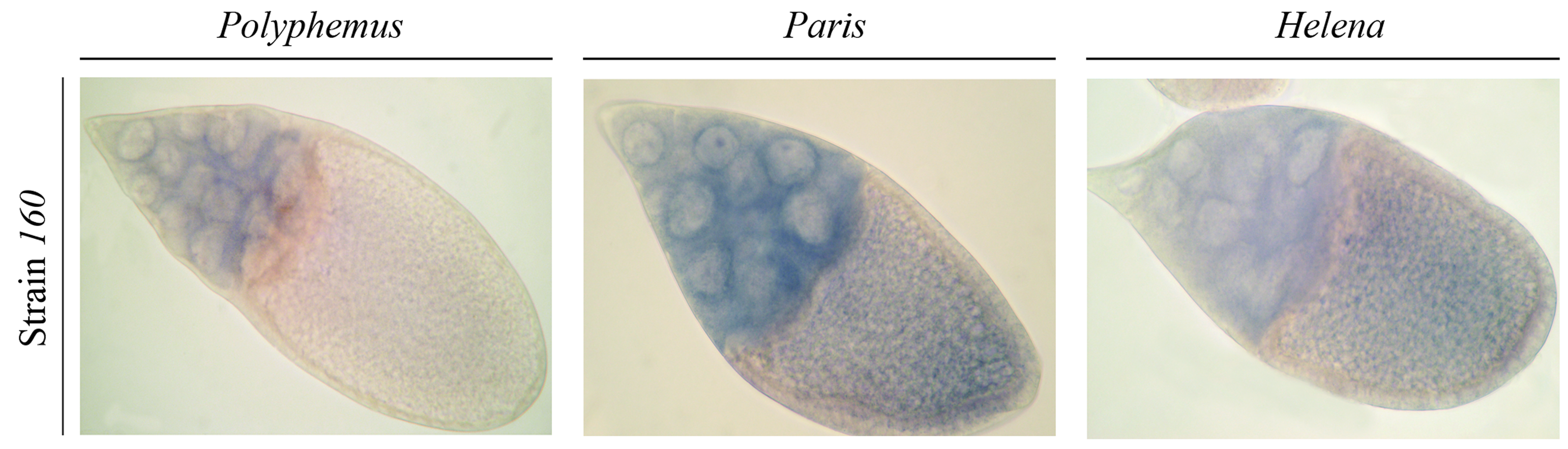

Supplement: S3 Fig — (TIF) [file pgen.1007400.s003.tif]

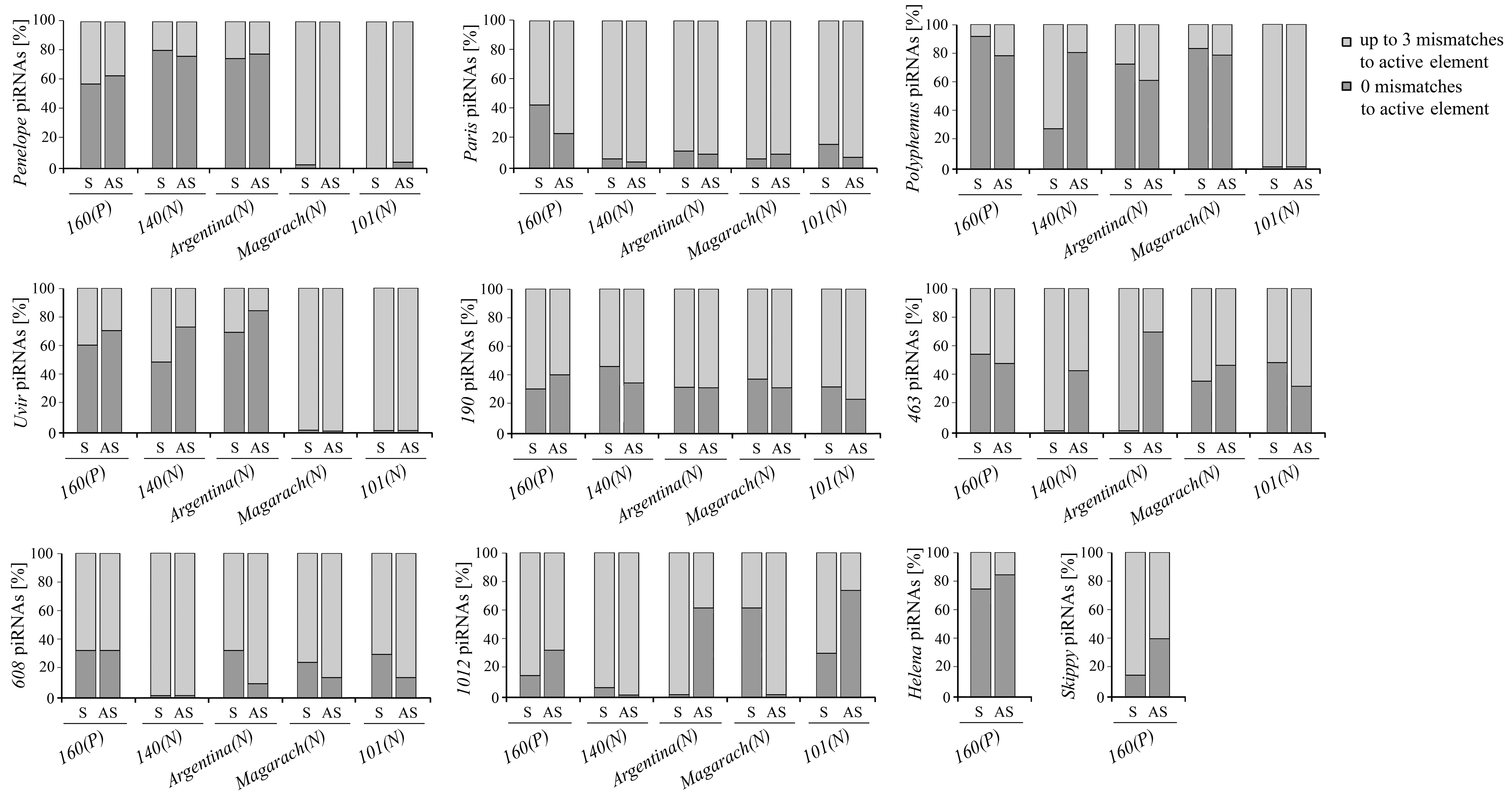

Supplement: S4 Fig — Number of mismatches (up to 3) to active element are indicated; piRNAs are split into sense (S) and antisense (AS) species. (TIF) [file pgen.1007400.s004.tif]

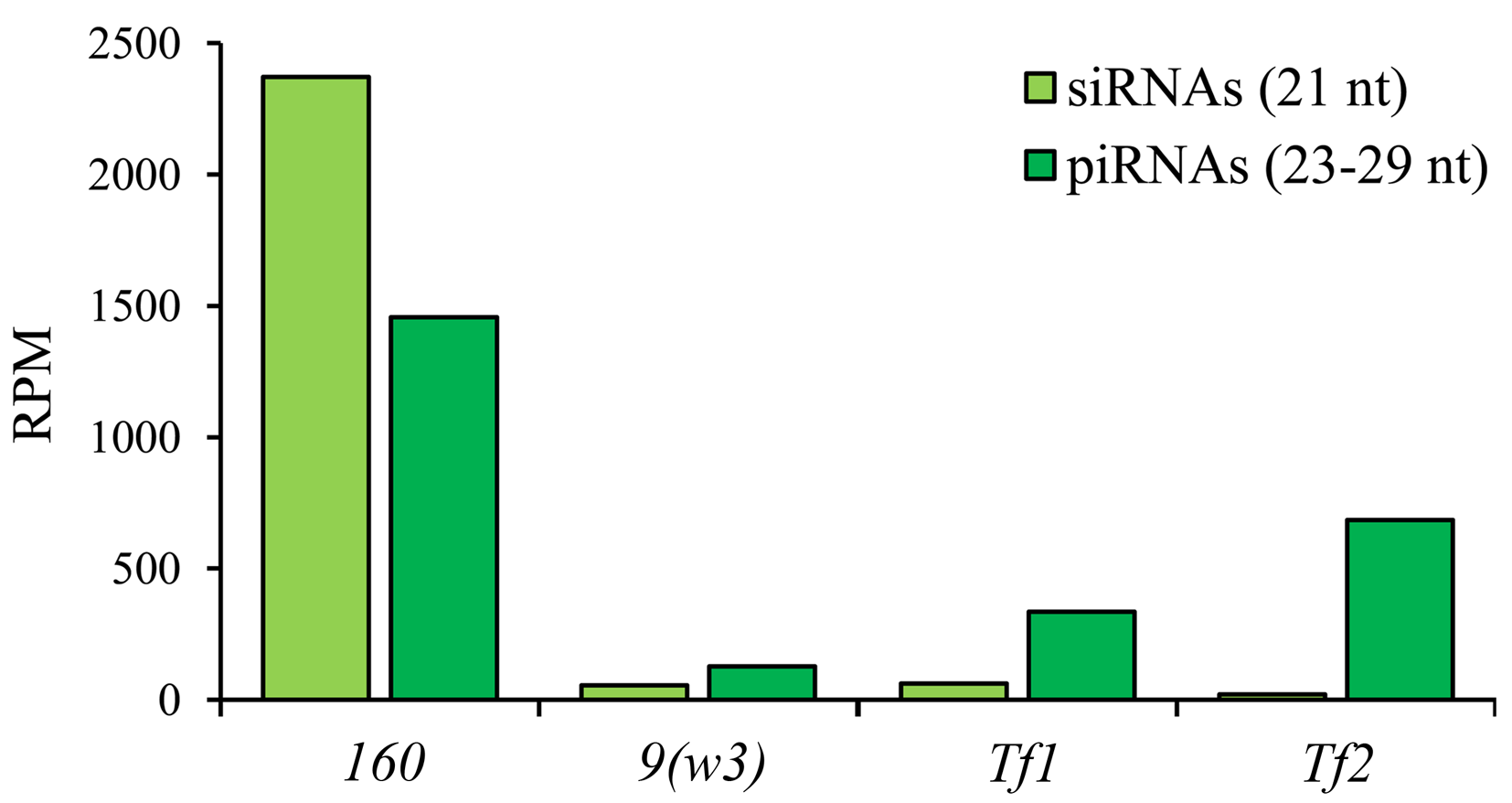

Supplement: S5 Fig — (TIF) [file pgen.1007400.s005.tif]

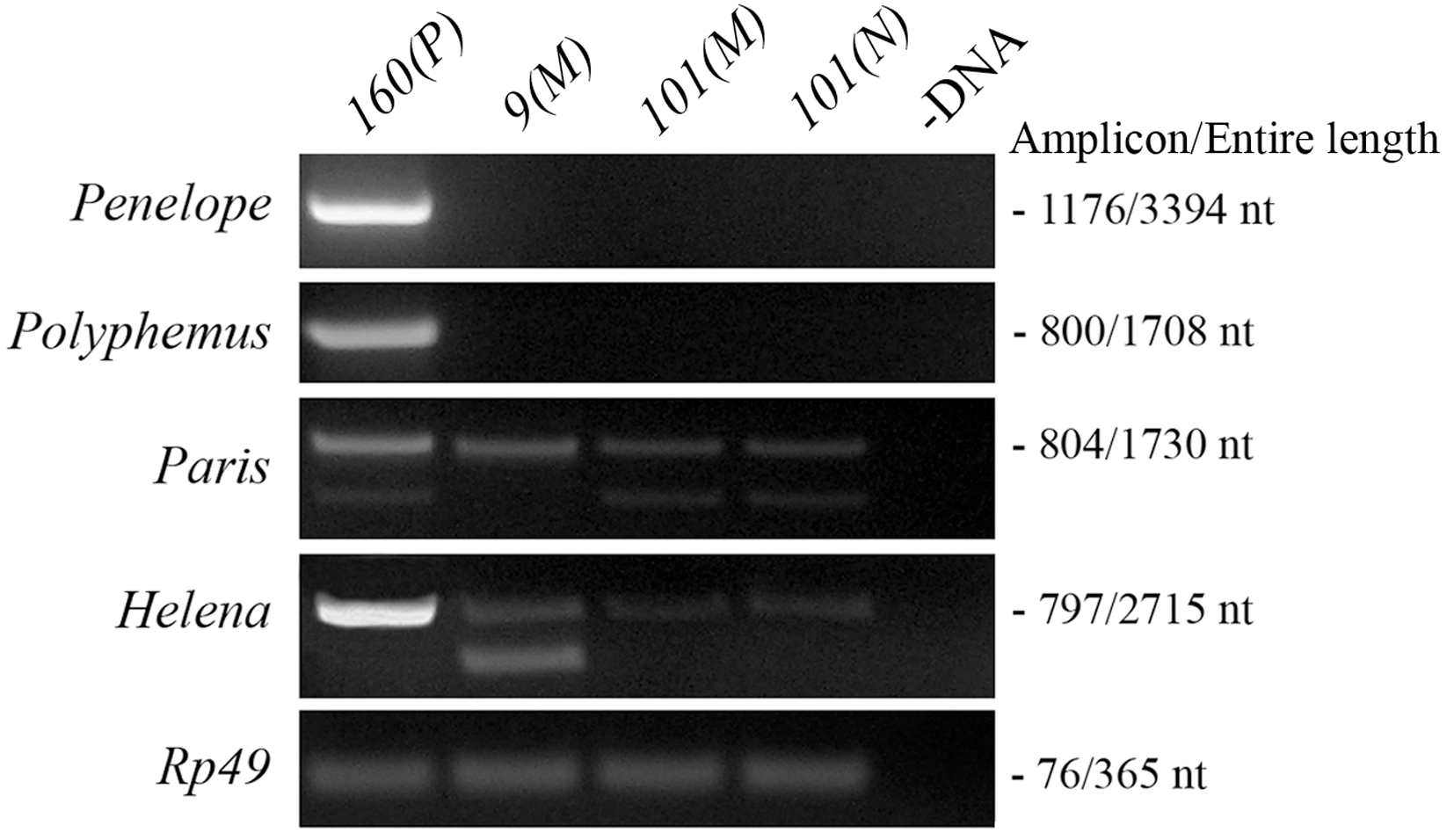

Supplement: S6 Fig — Primers correspond to the middle region of indicated TEs. Rp49 gene serves as a loading control. (TIF) [file pgen.1007400.s006.tif]

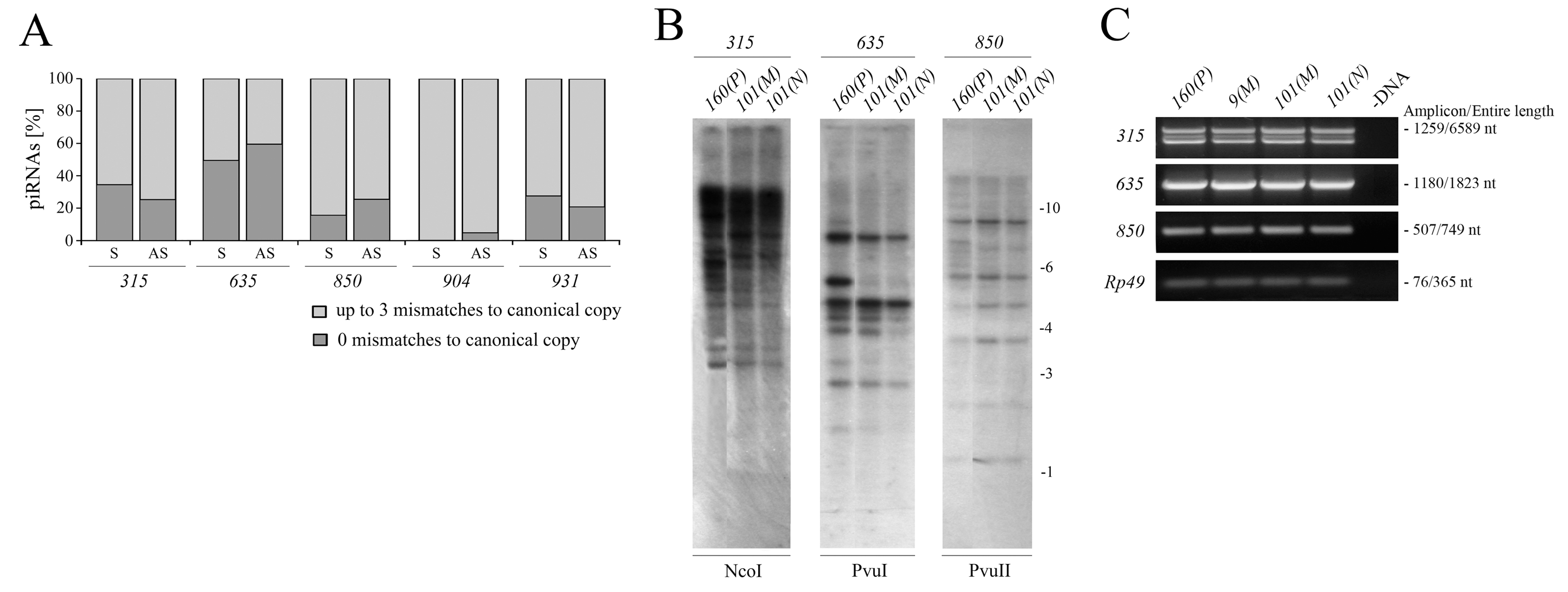

Supplement: S7 Fig — A) Mapping of 315, 635, 850, 904 and 931-piRNAs to canonical sequence of the element in strain 101(N). B) Southern-blot analysis of genomic DNA of 160(P), 101(M) and 101(N) strains. C) Semiquantitative PCR of 315, 635 and 850 elements of genomic DNA of 160(P), 101(M) and 101(N) strains. Primers correspond to the middle region of indicated TEs. Rp49 gene serves as a loading control. (TIF) [file pgen.1007400.s007.tif]

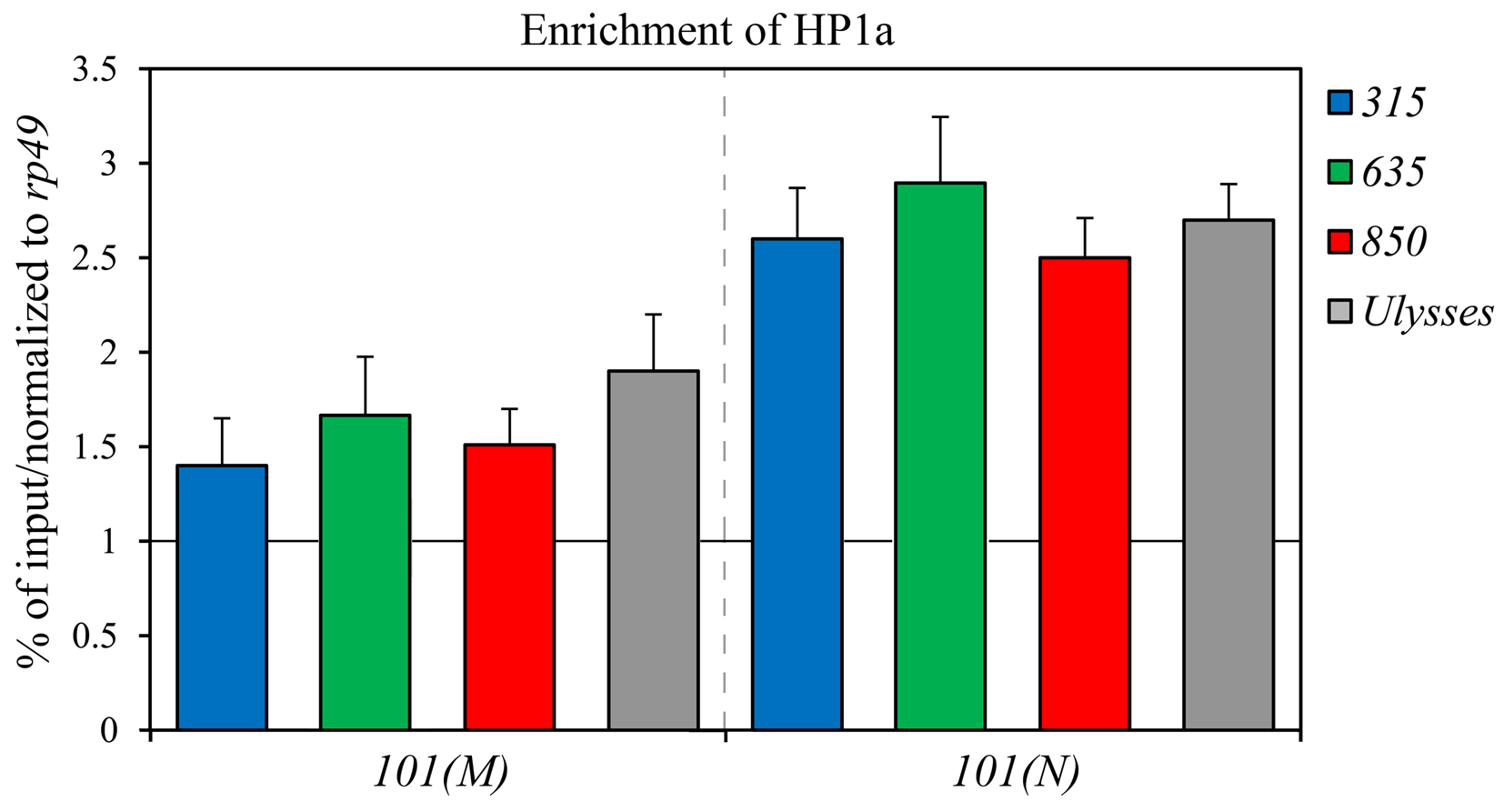

Supplement: S8 Fig — (TIF) [file pgen.1007400.s008.tif]

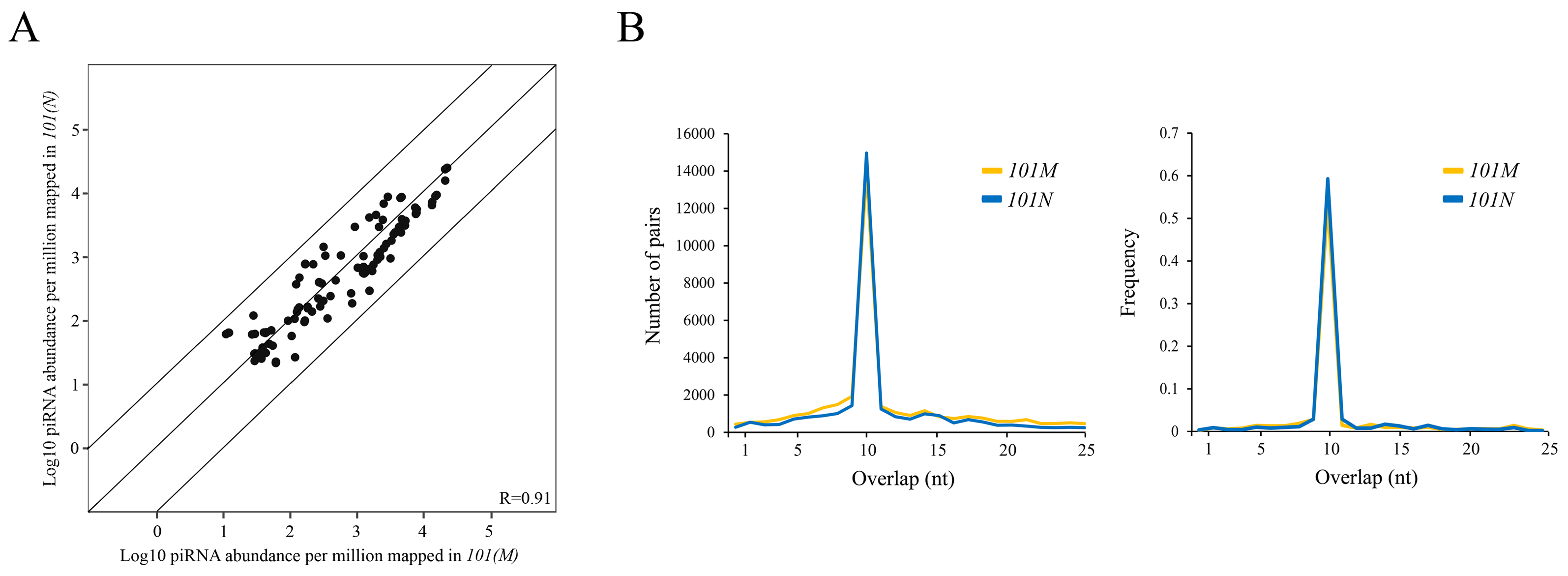

Supplement: S9 Fig — A) Scatter plot represents the result of comparison of normalized piRNAs (23–29 nt) in 101M and 101N strains. Diagonal lines indicate 10-fold levels of difference. The results of Spearman’s correlation tests (R) are demonstrated. B) At the left–number of consitituted ping-pong pairs; at the right–frequency of overlapped piRNAs. (TIF) [file pgen.1007400.s009.tif]

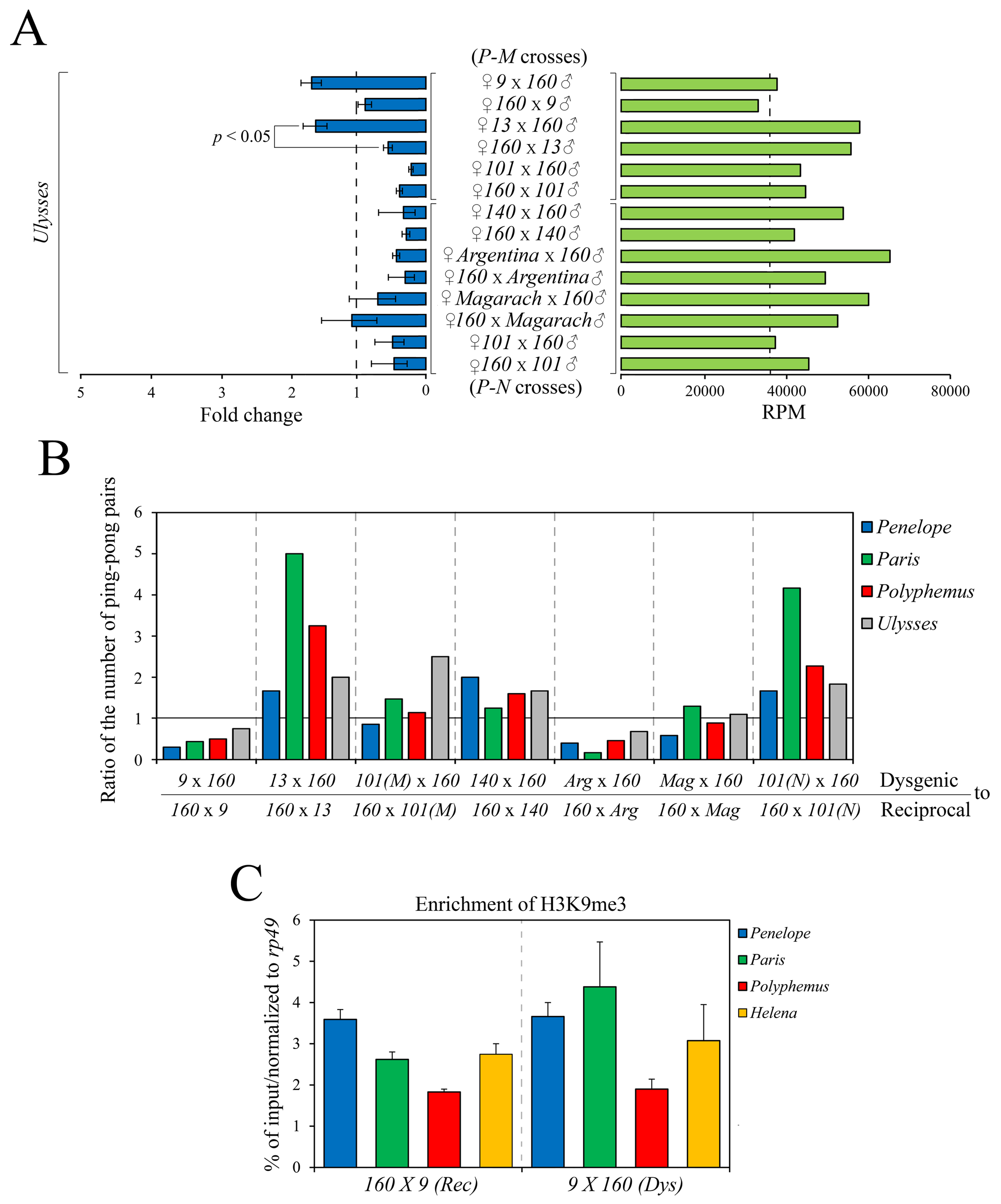

Supplement: S10 Fig — A) mRNA and piRNAs expression levels in the ovaries of the progeny from dysgenic and reciprocal hybrids. At the left—expression levels of indicated TEs relative to the level in P-strain 160. At the right–normalized piRNAs expression levels. The dotted line indicates level in P-strain 160. P-values were calculated using t-test. B) The ratio of constituted ping-pong pairs between the dysgenic and reciprocal hybrids. C) H3K9me3 ChIP-qPCR on ovaries of dysgenic and reciprocal hybrids involving P-strain 160 and M-like strain 9. (TIF) [file pgen.1007400.s010.tif]

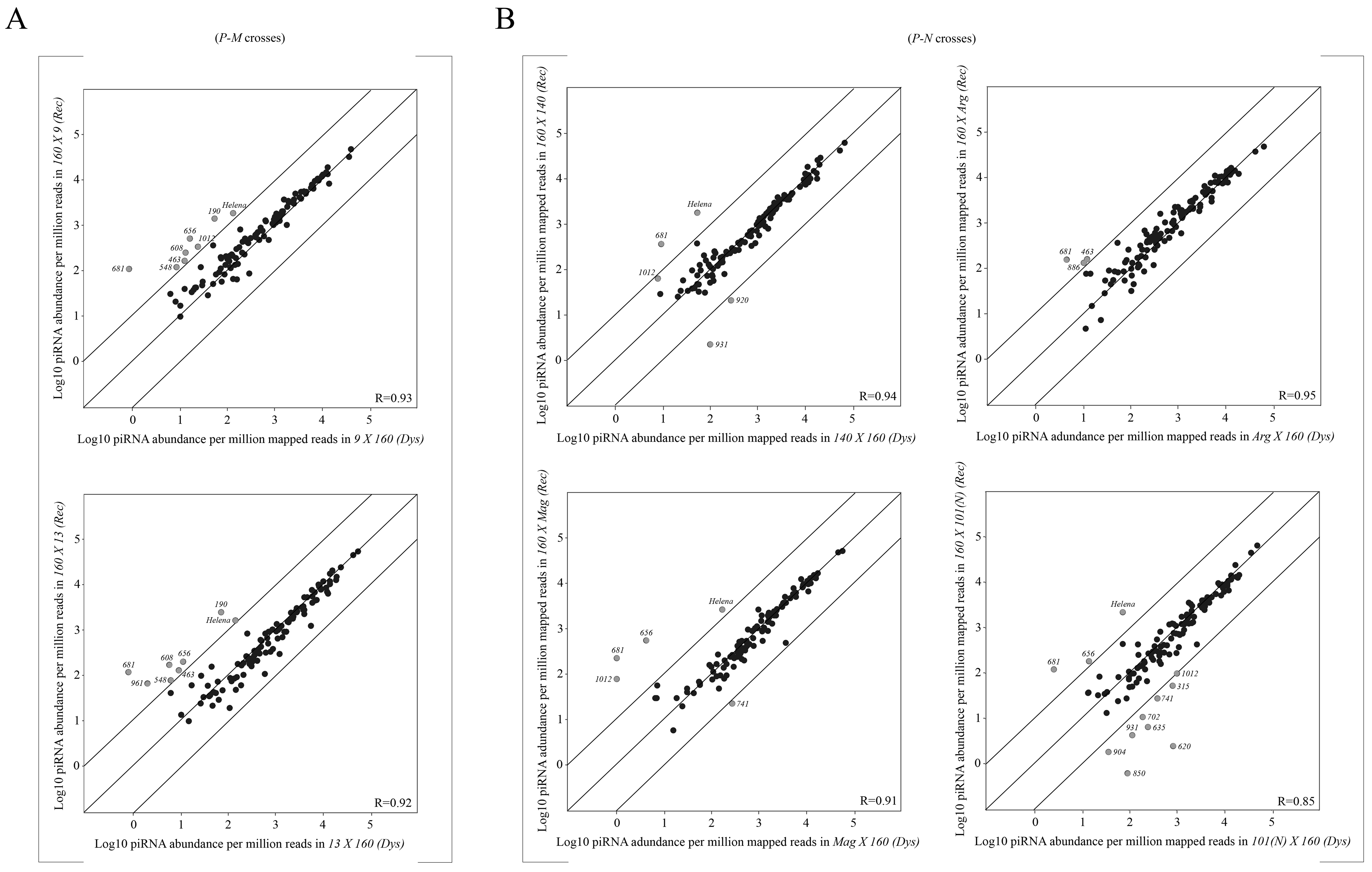

Supplement: S11 Fig — A) and B) Scatter plots represent the result of pairwise comparison of normalized piRNAs (23–29 nt) between dysgenic and reciprocal ovaries of strain 160 and M-strains and 160 and N-strains, respectively. Diagonal lines indicate 10-fold levels of difference. All the TEs that exceed 10-fold line are marked as gray dots. The results of Spearman’s correlation tests (R) are shown. (TIF) [file pgen.1007400.s011.tif]
